# Differential Response of Lung Cancer Cells, with Various Driver Mutations, to Plant Polyphenol Resveratrol and Vitamin D Active Metabolite PRI-2191

**DOI:** 10.3390/ijms22052354

**Published:** 2021-02-26

**Authors:** Ewa Maj, Beata Maj, Klaudia Bobak, Michalina Gos, Michał Chodyński, Andrzej Kutner, Joanna Wietrzyk

**Affiliations:** 1 Hirszfeld Institute of Immunology and Experimental Therapy, Polish Academy of Sciences, 12 Weigla, 53-114 Wrocław, Poland; maj.beata9@gmail.com (B.M.); klaudia.bobak13@gmail.com (K.B.); michalina.gos104@gmail.com (M.G.); joanna.wietrzyk@hirszfeld.pl (J.W.); 2 Łukasiewicz Research Network—Industrial Chemistry Institute, 8 Rydygiera, 01-793 Warsaw, Poland; m.chodynski@ifarm.eu; 3 Faculty of Mathematics and Natural Sciences, School of Sciences, Institute of Chemical Sciences, Cardinal Stefan Wyszyński University in Warsaw, 1/3 Wóycickiego, 01-938 Warsaw, Poland; a.kutner@uksw.edu.pl

**Keywords:** anticancer activity, lung cancer, resveratrol, PRI-2191, vitamin D

## Abstract

Plant polyphenols and vitamins D exhibit chemopreventive and therapeutic anticancer effects. We first evaluated the biological effects of the plant polyphenol resveratrol (RESV) and vitamin D active metabolite PRI-2191 on lung cancer cells having different genetic backgrounds. RESV and PRI-2191 showed divergent responses depending on the genetic profile of cells. Antiproliferative activity of PRI-2191 was noticeable in EGFRmut cells, while RESV showed the highest antiproliferative and caspase-3-inducing activity in KRASmut cells. RESV upregulated p53 expression in wtp53 cells, while downregulated it in mutp53 cells with simultaneous upregulation of p21 expression in both cases. The effect of PRI-2191 on the induction of CYP24A1 expression was enhanced by RESV in two KRASmut cell lines. The effect of RESV combined with PRI-2191 on cytokine production was pronounced and modulated. RESV cooperated with PRI-2191 in regulating the expression of IL-8 in EGFRmut cells, while OPN in KRASmut cells and PD-L1 in both cell subtypes. We hypothesize that the differences in response to RESV and PRI-2191 between EGFRmut and KRASmut cell lines result from the differences in epigenetic modifications since both cell subtypes are associated with the divergent smoking history that can induce epigenetic alterations.

## 1. Introduction

**Figure 1:**
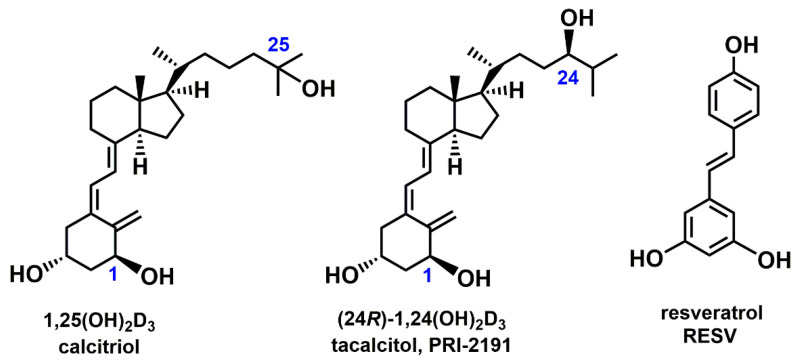
The chemical structure of 1,25(OH)_2_D_3_ (calcitriol), (24R)-1,24(OH)_2_D_3_ (PRI-2191, tacalcitol), and resveratrol (RESV).

Accumulating evidence has demonstrated the chemopreventive and therapeutic potential of polyphenolic antioxidants derived from plants in various preclinical models of human cardiovascular, metabolic, neurodegenerative, and cancer diseases. The chemopreventive and therapeutic effects of these compounds are believed to involve the regulation of signaling pathways like mitogen-activated protein kinases, p53-MDM2 (MDM—ubiquitin E3 ligase of the tumor suppressor p53), nuclear factor-kappaB (NFκB). By modulating these cell signaling pathways, polyphenols activate the cell death signals and induce apoptosis of precancerous or malignant cells, thereby inhibiting the development or progression of cancer [1,2,3]. In addition, the antiangiogenic activity of these compounds was under investigation. Studies showed that this activity of the plant polyphenols involves the inhibition of endothelial cell proliferation and migration, prevention of sprout formation, inhibition of matrix metalloproteinases, and modulation of angiogenic signaling pathways [4]. Commonly studied polyphenols, such as curcumin, quercetin, and resveratrol (RESV; Figure 1), interact with multiple protein targets and thus modulate the signaling pathways related to various diseases [5].

Plant-originated substances demonstrated synergism when used in combination with different anticancer agents. For example, RESV, a natural stilbenoid phenol, which is produced by plants in response to injury or attack of pathogens, was tested in combination with other substances, including anticancer drugs. Although there is a lack of randomized controlled trials analyzing the effect of this substance on humans, RESV sensitized tumor cells in neuroblastoma, glioma, breast cancer, prostate cancer, pancreatic cancer, and leukemia to the proapoptotic effects of cytostatics such as doxorubicin, 5-fluorouracil, or cisplatin [6]. Kubota et al. studied the activity of RESV and paclitaxel, one of the primary cytostatics used in the treatment of lung cancer. The authors reported the antiproliferative effect and pro-apoptotic properties of this cytostatic potentiated by RESV in lung A549 cells [7]. Baatout et al. demonstrated high-dose RESV sensitizing chronic myeloid leukemia or cervical cancer cells to X-rays [8]. Beneficial role of polyphenols in the prevention and management of lung cancer in vitro have been already described [9,10].

Moreover, 1,25-dihydroxycholecalciferol (1,25(OH)_2_D_3_, calcitriol; Figure 1), the most active hormonal form of vitamin D_3_, exerts anticancer effects by regulating proliferation, differentiation, apoptosis, and angiogenesis [11]. The antiproliferative activity of vitamin D compounds was therefore tested in different cancer models, including leukemia and lymphoma, breast cancer, prostate cancer, colon cancer, and lung cancer [12,13,14,15]. Studies on animal cancer models have also indicated several vitamin D analogs as potent agents, especially when used in combination with chemotherapeutics [16,17,18,19,20]. However, not all types of cancer cells are equally sensitive to the anticancer properties of vitamins D. Therefore, several studies were performed to understand why cancer cells vary in their response to vitamins D and what is the mechanism of the resistance of certain cancer cells to the antiproliferative activity of 1,25(OH)_2_D_3_ and other vitamin D compounds [21].

A number of oncogenic driver mutations have been identified in lung cancer with the EGFR and KRAS mutations as the most prevalent and with significant clinical implications. Briefly, EGFR mutations are common in never smokers and respond to targeted therapy with the use of tyrosine kinase inhibitors (erlotinib and gefitinib), while KRAS mutations are associated with smoking history and are difficult to target. What is more, EGFR and KRAS mutations are mutually exclusive [22,23]. Additionally, among genetic abnormalities responsible for tumorigenesis of lung cancer are mutations of tumor suppressor gene TP53 [22,24,25].

In our previous study, we analyzed the antiproliferative activity of vitamin D_3_ active metabolite ((24R)-1,24-dihydroxycholecalciferol ((24R)-1,24(OH)_2_D_3_, PRI-2191, tacalcitol; Figure 1)) alone and in combination with anticancer drugs (tyrosine kinase inhibitors and cytostatics) in in vivo A549 lung cancer model and showed that PRI-2191 enhanced anticancer activity of the drugs [18,19]. Following, we evaluated the antiproliferative activity of PRI-2191 in a panel of lung cancer cell lines and found that the cells having different genetic backgrounds revealed differential responses to this metabolite. The most vulnerable to antiproliferative activity of PRI-2191 was EGFR-mutant HCC827 lung cancer cell line, while KRAS-mutant cell lines were less sensitive. However, despite the effect of vitamin D compounds on cell proliferation was weak in some cell lines, vitamin D compounds were transcriptionally active as assessed on increased CYP24A1 expression [14]. Thus, EGFR-mutant lung cancer cells are more sensitive to the anti-proliferative effects of vitamin D in contrast to KRAS-mutant lung cancer cells. Although no significant antiproliferative effect of vitamin D was observed in KRAS mutations, induction of CYP24 expression, typical of vitamin D, is observed in these cells. This suggests that VDR is active in these cells, but does not show antiproliferative activity. Probably VDR is not able to activate the expression of these genes, which could contribute to the antiproliferative effect. Therefore, we decided to find out what is the difference in the response of both subtypes of lung cancer cells to the anti-cancer effects of vitamin D. We assume that, first, some other genes may also be expressed in KRASmut cells after vitamin D treatment, and second, the use of vitamin D when combined with another active substance like RESV, will respond differently to vitamin D, because some additional signaling mechanism or pathway will be activated. In the AML model, vitamin D induction of leukemia cell differentiation was shown to be potentiated by plant polyphenols. So, we decided to investigate the interaction of the active vitamin D metabolite with RESV as a plant polyphenol. Since these two cell subtypes responded differently to the vitamin D metabolite used, we were also interested in whether the response of the cells would be similar or different when RESV worked alone, and whether any relationship could be observed. Here, we asked the question, whether lung cancer cell lines of different origin respond similarly to anticancer properties of RESV and whether any correlation can be found between the mode of RESV action and the lung cancer cell type. Therefore, in this study, we aimed to analyze the influence of RESV on cell proliferation inhibition, cell cycle, apoptosis, and expression of some cytokines and proteins. Additionally, the activity of natural compounds can be modulated when used in combinations. The study on acute myeloid leukemia (AML) cell lines, representing different stages of myeloid maturation, showed that the differentiation-inducing activity of vitamin D analogs can be enhanced by combination with plant polyphenol carnosic acid [26]. Therefore, we decided to examine the activity of the combination of plant polyphenol RESV with an active metabolite of vitamin D PRI-2191 in a lung cancer model, that we previously tested in lung cancer cell lines [14], to see whether the activity of both compounds influences one another.

## 2. Results

### 2.1. Antiproliferative Activity of RESV and PRI-2191 in Lung Cancer Cell Lines

First, we determined the antiproliferative activity of RESV and PRI-2191 used alone on a panel of lung cancer cell lines briefly characterized in Materials and Methods. Cells were incubated with RESV and PRI-2191 by 72 h, and after that time, antiproliferative activity was assessed and inhibitory concentration 50 (IC_50_) was calculated. RESV showed antiproliferative activity against all the lung cancer cell lines tested in the study, but with differential potency. The highest activity was observed against NCI-H1581 and NCI-H1703 (IC_50_ 15–32 µM) and moderate activity against A549, NCI-H358, NCI-H1299, HCC827, and A-427 (IC_50_ 40–60 µM), while the weakest activity was observed against Calu-3 cells (IC_50_ above 200 µM) (Table 1). Treatment with PRI-2191 alone caused proliferation inhibition by 20.47% and 7.52% of HCC827 cells and by 11.5% and 8.82% in NCI-H1703 cells at the concentrations of 1000 and 100 nM, respectively. Cell proliferation was stimulated by 10.63% and 12.83% in NCI-H358 cells at the concentrations of 1000 and 100 nM, respectively (Table 2). However, PRI-2191 added at the concentration of 100 nM did not significantly improve the cytotoxic activity of RESV. Only in NCI-H1703 cells, a slight increase in the antiproliferative activity was observed after treatment with RESV in combination with PRI-2191 compared to that observed with RESV alone. On the contrary, in the case of NCI-H358, a decrease in the antiproliferative activity was observed when PRI-2191 was used together with RESV (Table 1). The dose-response curves of RESV used alone and RESV used in combination with PRI-2191 are presented in the Supplementary Material (Figure S1).

### 2.2. Cell Cycle Analysis of Lung Cancer Cells Treated with RESV and PRI-2191

**Figure 2:**
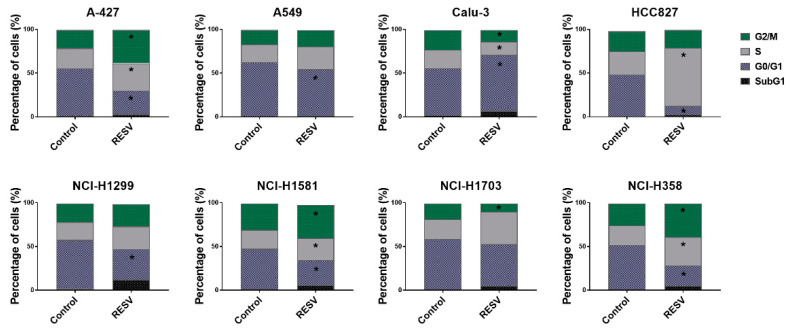
Flow cytometry cell cycle analysis of lung cancer cells after treatment with resveratrol (RESV) (20 μM). Data were analyzed using the Flowing Software v2.5.1. * Compared to control (untreated cells) (*p* < 0.05, Student’s *t*-test).

Next, we performed cell cycle analysis in lung cancer cells of different genetic backgrounds treated with RESV to check whether RESV activity is cell cycle dependent and whether it arrests cells in a given phase of cell cycle depending on the cell type or not. For this purpose, tested lung cancer cells were treated with RESV at the concentration of IC_25_–IC_35_ averaged to 20 µM. As shown in Figure 2, treatment with RESV resulted in diverse effects on cell cycle depending on the cell line. For instance, a decrease in the percentage of cells in the G0/G1 phase was observed for A-427, A549, HCC827, NCI-H1299, NCI-H1581, and NCI-H358. A simultaneous increase in cell percentage in the S phase was observed for HCC827 (the highest percentage of cells in S phase for these cells), A-427, NCI-H1581, and NCI-H358 cell lines (statistically significant), while a simultaneous increase in the percentage of cells in the G2/M phase was noticed for A-427, NCI-H1581, and NCI-H358. However, no changes in the percentage of cells in the G0/G1 phase were observed for NCI-H1703, but a small increase in the cell percentage in the S phase (not statistically significant) with a simultaneous significant decrease in the G2/M phase was noticed. Only for Calu-3 cells, RESV treatment resulted in an increase in the cell percentage in the G0/G1 phase with a concomitant significant decrease in the S and G2/M phase (Figure 2). The representative histograms of this analysis are presented in the Supplementary Material (Figure S2). The treatment with RESV alone or in combination with PRI-2191 caused a significant increase in the percentage of cells with fractional DNA content (sub-G1 cells) in the case of NCI-H1299, NCI-H1581, NCI-H1703, and NCI-H358 cell lines (for NCI-H1703, only the difference caused by treatment with RESV plus PRI-2191 was statistically significant) (Figure 2 and Figure S3).

When used alone, PRI-2191 did not influence the cell cycle of lung cancer cell lines. The exception was the cell line HCC827, for which PRI-2191 treatment resulted in an increased percentage of cells in the G0/G1 phase of the cell cycle compared to the non-treated cells (data showed in the Supplementary Material, Figure S3). A small reversal trend was noted in the effect of RESV with the addition of PRI-2191 in A549 and NCI-H358 cell lines. With the use of RESV alone, the increase observed in the percentage of cells in the S phase for NCI-H358 and the decrease observed in the G0/G1 phase for A549 were statistically significant, while the changes observed with the combination of RESV and PRI-2191 were not significant. In addition, a significant difference in the percentage of cells in the G0/G1 phase was observed for NCI-H358 cells treated with RESV combined with PRI-2191 compared to treatment with RESV alone (Figure S3).

### 2.3. Induction of Caspase-3 Activity by RESV and PRI-2191

**Figure 3:**
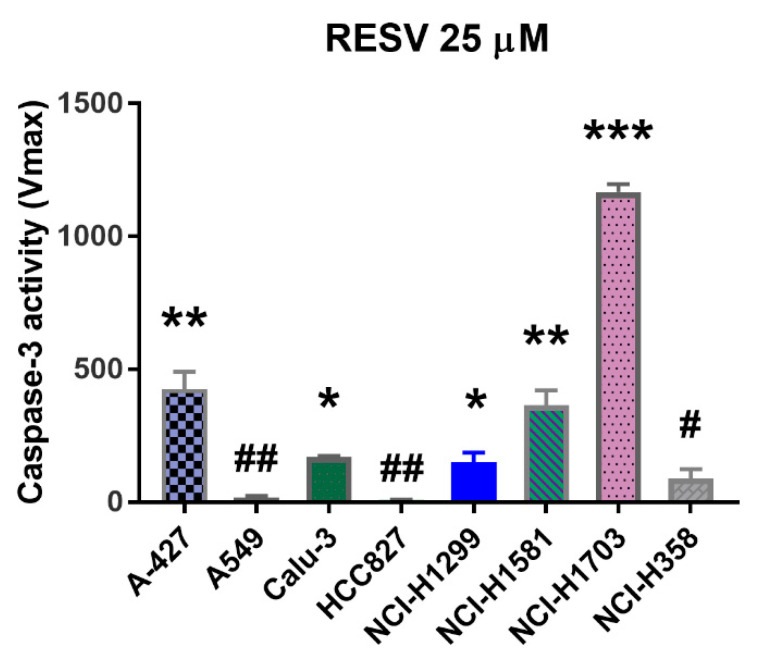
Induction of caspase-3 activity in lung cancer cells by RESV. RESV was used at a concentration of 0–75 µM and PRI-2191 at 100 nM. The above chart shows only the results for RESV at the concentration of 25 µM, while the remaining data (for 0–75 µM and 100 nM of PRI-2191) are presented in Supplementary material, Figure S4. Cells were lysed and the substrate (Ac-DEVD-ACC) was added to cell lysates. Fluorescence was measured with time, and kinetics was calculated as RFU/min. Data were analyzed using Gen5 2.09 software. * compared to A-427, A549, HCC827, and NCI-H1581, NCI-H1703; **compared to A549, Calu-3, HCC827, NCI-H1299, NCI-H1703, and NCI-H358; *** compared to all other cell lines; #compared to A-427, NCI-H1581, and NCI-H1703; ## compared to A-427, Calu-3, NCI-H1299, NCI-H1581, and NCI-H1703 (*p* < 0.05, one-way ANOVA with Tukey’s post hoc with multiple comparisons).

We analyzed also the activity of caspase-3 in lung cancer cells treated with RESV to check whether proliferation inhibition of lung cancer cells was a result of caspase-3-dependent cell death and to see if the ability of RESV to induce caspase activity was similar or not in tested lung cancer cells. Caspase-3 is a key executioner of caspases, which, in addition to caspase-7, is necessary for apoptosis. We used a method based on the enzyme’s ability to hydrolyze the synthetic Ac-DEVD-ACC substrate, upon treatment with RESV or PRI-2191 or both, which leads to the release of 7-amino-coumarin fluorochrome. Following the treatment, we measured the increase of fluorescence with time. RESV most effectively induced caspase-3 activity in NCI-H1703 cells (V_max_ = ~1000), while its activity was mild in A-427, Calu-3, and NCI-H1581 (V_max_ = ~400) and moderate in A549, NCI-H1299, and NCI-H358 (V_max_ = 100–200 in a concentration-dependent manner). The least caspase-inducing effect was found in EGFR-mutant HCC827 cells (V_max_ = 10–20) (Figure 3), which suggests that EGFR-mutant lung cancer cells are possibly the least vulnerable to caspase induction by RESV. It was also observed that PRI-2191 decreased both the basal caspase-3 activity (significantly in NCI-H1581, NCI-H1703, and NCI-H358) and the RESV-induced caspase-3 activity (significantly in Calu-3 and HCC827 at the indicated concentrations) (Figure S4).

### 2.4. Changes in p53 and p21 Expression in Lung Cancer Cells after RESV and PRI-2191 Treatment

Even though we did not observe the robust effect of combining RESV and PRI-2191, on anti-proliferative activity of lung cancer cells, in the following experiments, the impact of both RESV and PRI-2191, as well as their combination, on the expression of some proteins was tested. It could not be ruled out that some changes have taken place at the molecular level. Therefore, we have made an attempt to check this possibility on a few examples.

**Figure 4:**
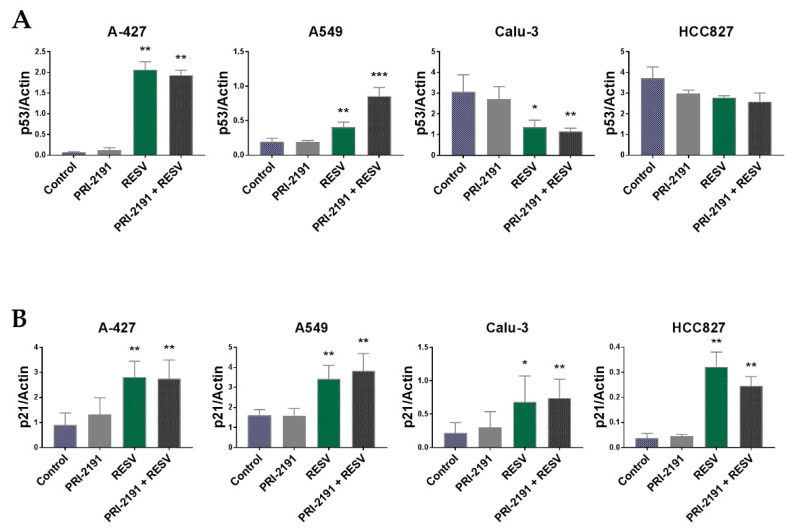
Western blot analysis of lung cancer cells treated with PRI-2191 (100 nM) and RESV (20 µM). Effect of PRI-2191 and RESV on (**A**) p53 and (**B**) p21 expression in lung cancer cells (all blots and statistical analysis for other cell lines are presented in Figure S6). Cell lysates were subjected to SDS-polyacrylamide gel electrophoresis and analyzed by Western blotting. Actin was used as a normalization control. * Compared to control (untreated cells); ** compared to control and PRI-2191; *** compared to control, PRI-2191, and RESV (*p* < 0.05, one-way ANOVA with Tukey’s post hoc with multiple comparisons).

We analyzed the expression of p53 and p21 proteins, which regulate the cell cycle progression and apoptosis, using the Western blot analysis. p53 is known also as the guardian of the genome and is more frequently mutated in human cancers than any other gene [25]. Here, the results revealed that RESV significantly induced the expression of p53 in A-427 and A549 cells (Figure 4), but only slightly in NCI-H1703 cells (not statistically significant in the latter) (Figure S6). Furthermore, the combination of PRI-2191 and RESV significantly augmented the upregulation of p53 in A549 cells compared to that observed with RESV alone (Figure 4). In Calu-3, the level of p53 was found to be significantly lowered after RESV treatment, while a decrease was also noted in HCC827, but it was not statistically significant (Figure 4). The expression of p21, which is regulated by p53, was upregulated simultaneously with p53 expression in A-427 and A549 cells. In addition, the level of p21 was found to be also increased in Calu-3 and HCC827 cells after treatment with RESV, although p53 expression was not upregulated. Similarly, the level of p21 in Calu-3 cells was also significantly upregulated with PRI-2191–RESV combination compared to that observed with PRI-2191 alone (Figure 4). Furthermore, RESV lowered the level of p21 in NCI-H1581 cells (Figure S6). No significant changes in p53 and p21 expression were observed for other tested cell lines (Figure S6).

RESV is mainly known to modulate the activity of SIRT1, a NAD+-dependent histone deacetylase [27,28]. However, SIRT1 is also responsible for the deacetylation of nonhistone proteins, such as p53 and VDR, and thus impact their activity [29,30]. Therefore, we estimated the level of SIRT1 expression to analyze whether it could be modulated by RESV and/or PRI-2191 in lung cancer cells. Western blot revealed the expression of SIRT1 in all lung cancer cell lines, but the treatment of cells with RESV and/or PRI-2191 did not significantly influence the level of expression (Figure S5).

### 2.5. Differential Expression of CYP24A1, RXRα, and VDR in Lung Cancer Cells after PRI-2191 and RESV Treatment

**Figure 5:**
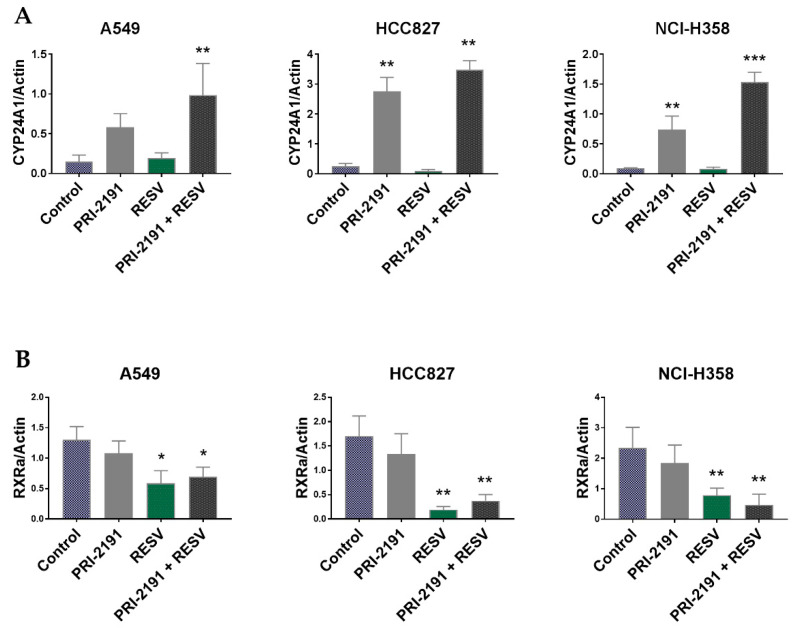

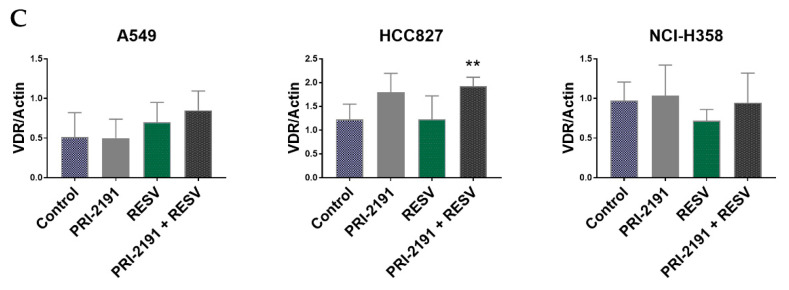
Western blot analysis of lung cancer cells treated with PRI-2191 (100 nM) and RESV (20 µM). Effect of PRI-2191 and RESV on (**A**) CYP24A1, (**B**) RXRα, and (**C**) vitamin D receptor (VDR) expression in lung cancer cells (all blots and statistical analysis for other cell lines are presented in Figure S7). Cell lysates were subjected to SDS-polyacrylamide gel electrophoresis and analyzed by Western blotting. Actin was used as a normalization control. * Compared to control (untreated cells); ** compared to control and RESV (for RXRα compared to control and PRI-2191); *** compared to control, RESV, and PRI-2191 (*p* < 0.05, one-way ANOVA with Tukey’s post hoc with multiple comparisons).

Afterwards, we analyzed the expression of the following key proteins that regulate the activity of vitamin D: VDR, CYP24A1 (24-hydroxylase, the enzyme responsible for vitamin D deactivation and the strongest known vitamin D-responsive gene), and RXRα (retinoid X receptor α, which together with VDR forms a heterodimer binding, e.g., to the promoter sequence of the CYP24A1) [31], to check whether their expression was modulated by RESV and PRI-2191 in lung cancer cells. Western blot analysis showed that the expression of CYP24A1 was significantly upregulated upon PRI-2191 treatment in Calu-3, HCC827, NCI-H358 cells, and only slightly and not statistically significantly in A-427 (also in RESV-treated cells), A549, and NCI-H1299 cells (Figure 5 and Figure S7). CYP24A1 expression was also significantly augmented by the PRI-2191–RESV combination in A549, HCC827, and NCI-H358 cells, compared to control cells and RESV-treated cells, and the expression was also significantly increased in NCI-H358 cells compared to PRI-2191-treated cells (Figure 5). The analysis of RXRα expression showed that treatment with RESV, either alone or in combination with PRI-2191, significantly upregulated the expression of this receptor only in A-427 (Figure S7), but downregulated the expression in A549, HCC827, NCI-H1703, and NCI-H358 cells. The expression was also downregulated in Calu-3 cells, but the difference was significant only when RESV was used with PRI-2191 (Figure 5 and Figure S7). The analysis of VDR expression showed a significant increase in the level only in NCI-H1299 lung cancer cells after treatment with PRI-2191 and when PRI-2191 was used with RESV (Figure S7). In addition, VDR expression was slightly upregulated in Calu-3 and HCC827 cells treated with PRI-2191 alone, but the increase was not statistically significant (Figure 5 and Figure S7). However, when PRI-2191 was used in combination with RESV, the level of VDR in the cells was increased in a statistically significant manner. Furthermore, the use of RESV also upregulated VDR expression to some extent in A549 cells, but the increase was not statistically significant in the replicates (Figure 5). Taken together, although in some cell lines the RESV treatment caused a decrease in the expression of RXRα, which cooperates with VDR in gene regulation, it did not influence the ability of PRI-2191 to induce CYP24A1 expression when both compounds were used together. Moreover, some improvement was observed in the ability of PRI-2191 to influence CYP24A1 expression with the addition of RESV.

### 2.6. Impact of RESV and -2191 on VEGF, PD-L1, IL-8, and OPN Expression

We analyzed the impact of RESV and PRI-2191 on the expression (at mRNA and protein level) of the following proteins regulating the processes responsible for cancer development: (a) vascular endothelial growth factor (VEGF) engaged in angiogenesis; (b) PD-L1 (also known as CD274 or B7-H1) responsible for tumor immune escape; (c) interleukin 8 (IL-8, also known as CXCL8), which is a multifunctional inflammatory chemokine produced by many cell types; and d) osteopontin (OPN, also known as SPP1), a multifunctional protein, highly expressed in bone, but also regulates immune cell functions, and whose expression is stimulated by 1,25(OH)_2_D_3_. The following cell lines were used for this analysis: A549 (KRASmut, p53wt), HCC827 (EGFRmut, p53mut), and NCI-H358 (KRASmut, p53null) in order to compare the activity of RESV and PRI-2191 on cells representing two main molecular subtype of lung cancer: EGFR mutant and KRAS mutant. Of the three, HCC827 was the most sensitive to the antiproliferative activity of PRI-2191, while the proliferation of NCI-H358 was stimulated by PRI-2191. In all three cell lines, CYP24A1 expression was found to be induced when PRI-2191 was used alone or in combination with RESV. The effect of RESV, PRI-2191, and their combination on the secretion of VEGF, OPN, and IL-8 by A549, HCC827, and NCI-H358 tumor cells was tested using enzyme-linked immunosorbent assay (ELISA) in conditioned medium, while the level of PD-L1 was analyzed in cell lysates.

**Figure 6:**
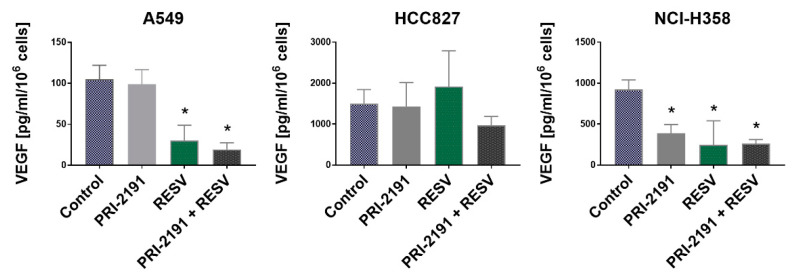
Effect of PRI-2191 and RESV on vascular endothelial growth factor (VEGF) secretion by lung cancer cells. RESV was used at a concentration of 20 μM and PRI-2191 at 100 nM. Cells were exposed to tested compounds for 72 h, then washed with PBS, and cultured in a serum-free medium for the next 24 h. Conditioned medium was collected, and the level of VEGF secreted by lung cancer cells was measured by ELISA. A549: * compared to control (untreated cells) and PRI-2191; NCI-H358: * compared to control (*p* < 0.05, one-way ANOVA with Tukey’s post hoc with multiple comparisons).

RESV, when used alone or in combination with PRI-2191, caused a significant decrease in the secretion of VEGF by A549 and NCI-H358 cells. Besides, a significant reduction in VEGF secretion was noted in NCI-H358 cells after treatment with PRI-2191 alone. By contrast, RESV did not affect the secretion of VEGF by HCC827 cells (Figure 6). Also, RESV appear to reveal antiangiogenic activity in KRAS-mutant lung cancer, but not in EGFR mutant. No significant changes in the expression of VEGF mRNA were observed between the treatment groups (Figure S9). 

**Figure 7:**
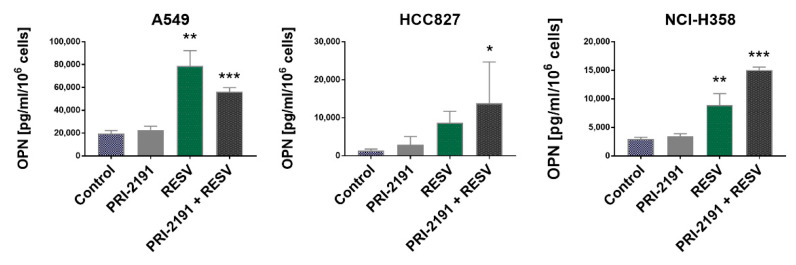
Effect of PRI-2191 and RESV on osteopontin (OPN) secretion by lung cancer cells. RESV was used at a concentration of 20 μM and PRI-2191 at 100 nM. Cells were exposed to tested compounds for 72 h, then washed with PBS, and cultured in a serum-free medium for the next 24 h. Conditioned medium was collected, and the level of OPN secreted by lung cancer cells was measured by ELISA. * Compared to control; ** compared to control (untreated cells) and PRI-2191; *** compared to control, PRI-2191, and RESV (*p* < 0.05, one-way ANOVA with Tukey’s post hoc with multiple comparisons).

Analysis of OPN secretion by lung cancer cells showed that RESV, when used either alone or in combination with PRI-2191, significantly increased the level of this protein in conditioned medium of A549 and NCI-H358 cells, while this effect was observed only with RESV–PRI-2191 combination in HCC827 cells. On the other hand, the addition of PRI-2191 to RESV caused an opposite effect on A549 and NCI-H358 cells; a significantly lower level of OPN was found in A549 cells compared to those treated with RESV alone, while the OPN level was significantly higher in NCI-H358 cells (Figure 7). Quantitative polymerase chain reaction (qPCR) revealed that the expression of OPN was significantly upregulated in lung cancer cells treated with RESV alone and RESV in combination with PRI-2191, and also in HCC827 cells treated with PRI-2191 alone (Figure S10). Therefore, we concluded that KRAS-mutant lung cancer cells were more receptive to modulation of OPN expression by RESV than EGFR-mutant lung cancer cells.

**Figure 8:**
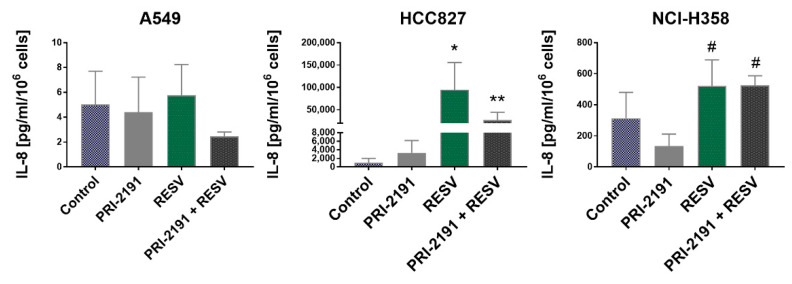
Effect of PRI-2191 and RESV on interleukin (IL-8) (CXCL8) secretion by lung cancer cells. RESV was used at a concentration of 20 μM and PRI-2191 at 100 nM. Cells were exposed to tested compounds for 72 h, then washed with PBS, and cultured in a serum-free medium for the next 24 h. Conditioned medium was collected, and the level of IL-8 secreted by lung cancer cells was measured by ELISA. * Compared to control (untreated cells) and PRI-2191; ** compared to RESV; # compared to PRI-2191 (*p* < 0.05, one-way ANOVA with Tukey’s post hoc with multiple comparisons).

Treatment with RESV resulted in a significant increase in the level of IL-8 of HCC827 cells compared to control cells, but the combination of PRI-2191 and RESV caused a significant reduction in the IL-8 level compared to cells treated with RESV alone (Figure 8). At the mRNA level, a significant increase in IL-8 expression was observed in HCC827 cells when they were treated with RESV alone (Figure S11). In the case of NCI-H358 cells, an increase in IL-8 level was observed using RESV, either alone or in combination with PRI-2191, but the difference was statistically significant compared to cells treated with PRI-2191 alone. A549 cells secreted a small basal amount of IL-8 compared to the other two cell lines. After treatment with RESV combined with PRI-2191, a slight decrease in IL-8 level was observed, but the difference was not statistically significant (Figure 8). Therefore, RESV treatment impacts IL-8 expression only in EGFR-mutant lung cancer cells.

**Figure 9:**
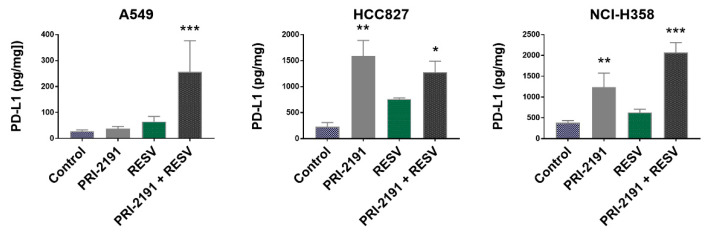
Effect of PRI-2191 and RESV on programmed death-ligand 1 (PD-L1) expression by lung cancer cells. RESV was used at a concentration of 20 μM and PRI-2191 at 100 nM. Cells were exposed to tested compounds for 72 h, then washed with PBS, and cultured in a serum-free medium for the next 24 h. Cells were collected, and the level of PD-L1 in lung cancer cells was measured by ELISA. * Compared to control (untreated cells); ** compared to control and RESV; *** compared to control, PRI-2191, and RESV (*p* < 0.05, one-way ANOVA with Tukey’s post hoc with multiple comparisons).

Analysis of PD-L1 expression in lung cancer cells showed that PRI-2191 significantly upregulated the expression of this molecule in HCC827 and NCI-H358 cells, while in A549 cells, an expression was increased only when PRI-2191 was used with RESV. Additionally, PRI-2191 combined with RESV caused a significant increase in PD-L1 expression in NCI-H358 cells compared to PRI-2191 used alone. RESV used alone also induced the expression of PD-L1 in HCC827 cells, but the increase was not statistically significant (Figure 9). Treatment with PRI-2191 and PRI-2191 in combination with RESV also significantly induced PD-L1 mRNA expression in HCC827 and NCI-H358 cells, while a significant induction in expression was observed for RESV alone or in combination with PRI-2191 in A549 cells (Figure S12).

Furthermore, the expression of SIRT1, VDR, and RXRα was analyzed at the mRNA level in all the chosen cell lines (A549, HCC827, and NCI-H358) utilizing qPCR, but no significant changes were observed after treatment with PRI-2191 and RESV (Figure S8).

## 3. Discussion

We evaluated the biological activity of RESV against lung cancer cells with different genetic background. The cell lines were chosen based on key mutations driving lung cancer: EGFR, KRAS, and additionally TP53 mutation status, in order to evaluate whether lung cancer cell lines of different origin respond similarly to anticancer properties of RESV and whether there is any correlation between the mode of RESV action and the lung cancer cell type. We also first combined the vitamin D active metabolite PRI-2191 with the plant polyphenol RESV and evaluated their biological effects on these lung cancer cell lines. This idea came out from the cross-talk between these two nutrients and also from observations that different plant polyphenols and vitamins D may advantageously cooperate in anticancer activity [26,32,33,34,35,36,37].

Initial in vitro antiproliferative activity assay of plant polyphenol RESV against lung cancer cells showed that RESV revealed the highest antiproliferative activity against NCI-H1703 cells and the activity was weakest against Calu-3 (the lowest and the highest IC_50_, respectively). However, when RESV and PRI-2191 were used in combination, no improvement in proliferation inhibition was found. On the other hand, in NCI-H358 cells, the addition of PRI-2191 to RESV caused a reduction in the antiproliferative activity of RESV.

Next, we tested the influence of RESV on cell cycle and the activity of caspase-3, an indicator of apoptosis and compared achieved results between tested cells. The ability of RESV to arrest the progression of cell cycle depending on the cancer cell line origin was reported. For example, the impact of RESV on cell cycle progression varied in prostate cancer cell lines, depending on their molecular subtype: androgen vs. estrogen receptor-expressing cells (LNCaP and PC-3, respectively) [38]. A cell-specific mechanism of cell cycle modulation and apoptosis induction by RESV was also reported in breast cancer cell lines MCF-7 and MDA-MB-231, which represent two molecular subtypes: estrogen receptor-positive and triple-negative, respectively [39]. In our study, we found that RESV, when used alone, promoted the accumulation of cells in the G0/G1 phase and S or G2/M phase depending on the lung cancer cell lines, with the most significant arrest in G2/M phase for HCC827 cell line, which is the representative of EGFR-mutant lung cancer. A small reversal trend was noted in the effect of RESV on cell cycle progression when PRI-2191 was added. The strongest induction of caspase-3 by RESV was observed in TP53-mutant lung squamous cell carcinoma NCI-H1703 cells, which may explain that these cells were the most sensitive ones to the antiproliferative activity of RESV (the lowest IC_50_ of RESV). The weakest ability of RESV to induce caspase-3 was noticed for EGFR-mutant lung adenocarcinoma HCC827 cells. In addition, PRI-2191 did not significantly inhibit the activation of caspase-3 induced by RESV, and only a decrease of this activity was observed. Taken together, the impact of RESV on proliferation inhibition, cell cycle progression, and apoptosis induction was different depending on the target lung cancer cells, but was not significantly or strongly modulated by the addition of PRI-2191. 

RESV and vitamin D are known to regulate signaling molecules including p53 protein, which contribute to cell cycle arrest as well as programmed cell death and DNA repair. In our study, we observed that RESV upregulated the expression of p53 in A-427 and A549 cells (both cell lines carry wtp53). The addition of PRI-2191 to RESV significantly augmented the upregulation of p53 only in A549 cells, which indicates the cooperative action of these two compounds in this cell line. On the other hand, a decline in the level of p53 was observed after RESV treatment in Calu-3 and HCC827 cells, both of which carry mutant p53 (M237I and V218del, respectively) according to the IARC TP53 Database. Ferraz da Costa et al. showed that RESV decreased the level of p53 mutant R248Q in HCC70, a highly invasive human breast ductal carcinoma cell line. By contrast, in MCF-7 cells, carrying wild-type p53, RESV increased the level of the protein [40]. It is already known that mutant p53 frequently loses its tumor-suppressive effect and gains new, undesirable oncogenic properties [41]. M237I, a mutant form of p53, has been shown to form amyloid oligomers in glioblastoma cells, which presented a chemoresistant gain-of-function phenotype [42]. Therefore, the downregulation of mutant p53 expression by RESV may be considered beneficial. Furthermore, Yi et al. showed that a 4-h treatment with the boronic acid chalcone analog of combretastatin A-4, YK-3-237, caused deacetylation of the mutant M237I-p53 at lysine 382 (K382) by activating the expression of SIRT1 in a triple-negative breast cancer. Deacetylation resulted in the depletion of the mutant p53 protein while upregulating the expression of the wild-type p53 target genes, such as PUMA and NOXA, which suggests that deacetylation leads to the reactivation of the wild-type p53 activity [43]. In this study, after treatment of Calu-3 and HCC827 cells with RESV alone or in combination with PRI-2191, we observed that p21 expression was reactivated. Willis et al. showed that mutant p53 exhibited a dominant-negative effect by preventing the wild-type p53 from inducing p21 expression [44]. Furthermore, by reducing the level of mutant p53 through treatment with RESV, the diminished induction of p21 was restored in lung cancer cells carrying mutant p53. On the other hand, it was speculated that acetylation of p53 at K120, K373, and K382 is crucial for the induction of p21 and the suppression of Ras-mediated tumorigenesis [45,46]. If we assume that RESV treatment deacetylated wild-type p53 through SIRT1 induction, then according to the reported data it can be expected that p53 was inactivated and its transcriptional activation of target genes, such as p21, was prevented. As shown here, in two RAS-mutant/p53-wild-type cell lines A-427 and A549 RESV treatment upregulated the expression of wild-type p53 and p21. Brochier et al. identified that K382 acetylation prevented the association of p53 with the proapoptotic gene promoter PUMA in mouse cortical neurons, highlighting that the consequence of p53 acetylation/deacetylation may be context-dependent [47]. Therefore, analyzing the acetylation status of p53 in lung cancer cells following RESV treatment could explain these differences in RESV activity on wild-type vs. mutant p53.

It was revealed that RESV and SIRT1 cooperate with vitamin D to enhance VDR signaling. Sabir et al. showed that SIRT1 and RESV potentiated the vitamin D-stimulated expression of CYP24A1 in HEK293 embryonic, kidney-derived cells [30]. On the other hand, we have previously shown that in lung cancer cells that were either sensitive or resistant to the antiproliferative activity of vitamin D, vitamin D signaling was active as revealed by the upregulated expression of CYP24A1 after treatment with vitamin D compounds [14]. In the present study, CYP24A1 expression was significantly upregulated upon PRI-2191 treatment in Calu-3, HCC827, and NCI-H358 cells, and when PRI-2191 was combined with RESV, a significant upregulation of expression was also noticed in A-427 and A549 cells. Further potentiation of CYP24A1 expression by RESV, when used with PRI-2191, was seen in NCI-H358. Therefore, in those cells, RESV cooperated with PRI-2191 in CYP24A1 expression. It should be noted that vitamin D itself directly influences the expression levels of VDR, increasing the mRNA level and stability of VDR, thus protecting against its degradation [48]. In this study, PRI-2191 used alone significantly induced VDR expression only in NCI-H1299 cells, but when combined with RESV, the receptor expression was significantly enhanced in Calu-3 and HCC827 cells while it was moderate in A549 cells. Thus, it may be concluded that depending on the cell line, RESV may influence vitamin D signaling in lung cancer cells, increasing the activity of vitamin D when combined with PRI-2191.

The effect of vitamin D is mainly mediated by VDR, which is a member of the steroid nuclear receptor superfamily. Vitamin D binds and activates VDR that functions as a transcription factor modulating the transcriptional activity of the vitamin D target genes. The binding of vitamin D results in conformational changes in VDR, facilitating the recruitment of its co-receptor, RXR. Vitamin D-liganded VDR–RXR heterodimer binds to vitamin D-responsive elements (VDREs) and regulates the expression of the target genes [49]. RESV potentiates the actions of 1,25(OH)_2_D_3_ by facilitating the heterodimerization of VDR with RXR, thus causing a cooperative effect on gene transactivation [50]. In our study, we observed that treatment with RESV resulted in the downregulation of RXRα expression in four out of eight cell lines and its upregulation in only one cell line. Wassermann et al. reported similar findings in the acute myeloid leukemia model for carnosic acid and silibinin. However, the study showed the opposite effect of silibinin on the prodifferential activity of vitamin D in myeloblastic HL60 and promonocytic U937 cells, accompanied by the upregulation of RXRα expression in HL60 cells and downregulation in U937 cells. This indicated that the modulation of RXRα by plant polyphenols is cell type specific [35]. A study on E12 embryos of diabetic dams showed that RESV modulated the expression of RXR in diabetic embryopathy and normalized the diabetes-induced suppression of the receptor level [51]. What is more, as it was showed in this study, although RESV decreased RXRα expression, it did not affect the ability of the PRI-2191 to induce CYP24A1 expression.

Several studies have examined the dual-anticancer effects of phytochemicals such as RESV and curcumin, combined with 1,25(OH)_2_D_3_. One example is the inhibition of tumor angiogenesis and augmentation of the antiproliferative and prodifferential activity of 1,25(OH)_2_D_3_. In the triple-negative breast cancer in vivo model, it was shown that RESV with 1,25(OH)_2_D_3_ reduced the vessel diameter of the tumor, and blood vessels of the combinatorial treatment group showed normal vessel morphology indicating vessel normalization unlike the groups receiving each agent alone. RESV also significantly induced endothelial cell death in vitro, which probably might be the reason for the reduced number of tumor microvessels found in the group receiving RESV and 1,25(OH)_2_D_3_ [52]. In the present study, we evaluated the ability of RESV and PRI-2191 to modulate the expression of VEGF in lung cancer cells. We found that only in both the KRAS-mutant lung cancer cell lines tested, RESV (alone or in combination with PRI-2191) decreased the level of secreted VEGF. Similarly, inhibition of VEGF production by RESV was also observed in human leukemia U937 cells, in stimulated human gingival fibroblasts, in A549 cells cocultured with adipose-derived mesenchymal stem cells, and c-FLIP-overexpressing H460 lung cancer cells [53,54,55,56]. Reinmuth et al. reported that VEGF expression correlated with EGFR mutational status in clinical specimens obtained from lung cancer patients. It was also shown that EGFR-mutant lung tumors showed a significantly higher VEGF expression than EGFR-wild-type tumors [57]. In HCC827, a lung cancer cell line with EGFR mutation, RESV either alone or in combination with PRI-2191 did not affect the level of VEGF secreted by the cells. Therefore, it may be concluded that RESV normalizes tumor angiogenesis in KRAS-mutant lung cancer but not in EGFR-mutant ones.

OPN is a protein that plays a key role in the progression and metastasis of several tumors, including lung, breast, prostate, or liver [58]. Overexpression of this protein in lung cancer has been shown to correlate with poor prognosis [59]. OPN promotes the epithelial–mesenchymal transition (EMT) in many types of cancer, such as breast or prostate cancer, as well as non-small cell lung cancer. In the present study, we tested the cooperative activity of RESV and PRI-2191 on the secretion of OPN, the expression of which is known to be regulated by 1,25(OH)_2_D_3_ [60,61]. We found that RESV increased the secretion of OPN in all three lung cancer cell lines examined. In particular, a significant increase in OPN secretion was noted for A549 and NCI-H358 cells, after incubation with RESV. RESV was also shown to induce OPN expression in mesenchymal bone marrow cells or human periodontal ligament cells. Furthermore, RESV synergized with 1,25(OH)_2_D_3_ in the induction of OPN expression in bone marrow osteoblast precursors [62,63]. Additionally, it was revealed that the incubation of periodontal ligament cells with the SIRT1 activator RESV increased the expression of differentiation markers, namely, alkaline phosphatase, OPN, and osteocalcin. It was also confirmed using siSIRT1 that SIRT1 stimulated osteoblastic differentiation [63]. Li et al. studied the role of SIRT1 in OPN-induced EMT in lung cancer cells A549 and NCI-H358 and revealed that the overexpression of SIRT1 attenuated EMT induction by OPN. While OPN decreased the expression of E-cadherin, upregulated the expression of N-cadherin and vimentin, and induced cell migration and invasion, SIRT1 overexpression prevented these effects. Moreover, exogenously added OPN caused the downregulation of SIRT1 expression in A549 and NCI-H358 cells [64]. Therefore, despite the induction of OPN expression by RESV, it could be expected that by activating SIRT1, RESV may counteract the protumorigenic effects of OPN. The opposite effects were noticed with the addition of PRI-2191 to RESV on OPN expression in A549 and NCI-H358 cells. PRI-2191 counteracted the induction of OPN expression in A549 cells but augmented the induction in NCI-H358 cells. The promoter region of the OPN gene contains a VDRE [60], and it is known that 1,25(OH)_2_D_3_ strongly induces OPN expression [62,65]. These together may explain the increase in OPN secretion by the addition of PRI-2191 to RESV in NCI-H358 cells. An interesting observation, however, is the lower level of OPN after treatment with RESV in combination with PRI-2191 observed in A549 cells, but the explanation of the mechanism responsible for it requires additional research.

RESV is also known for its anti-inflammatory properties since it inhibits the NFκB pathway. The activation of this pathway is required for the expression of many proteins involved in the inflammatory response, such as granulocyte-macrophage colony-stimulating factor, cyclooxygenase 2, inducible nitric oxide synthase, and IL-8 [66]. In the present study, we noticed a strong induction of IL-8 expression by RESV in the HCC827 cell line. Similar observations were made by Tino et al. in their study on the effect of RESV on VEGF and IL-8 expression in ovarian cancer cells [67]. Pastore et al. also showed that keratinocytes exposed to RESV displayed increased IL-8 expression. In addition, they noticed that RESV caused continuous activation of the EGFR signaling pathway. Therefore, the authors concluded that RESV induces delayed IL-8 expression through continuous activation of the EGFR-ERK pathway [68]. HCC827 cells tested in this study have an EGFR-activating mutation, which explains why these cells secreted the highest level of IL-8 compared to the other tested cell lines. In turn, Fan et al. showed that RESV inhibited EGFR phosphorylation only in gefitinib-resistant lung cancer cells (NCI-H1975), but not in the cells with wild-type EGFR (A549, NCI-H358). The authors suggested that the RESV analog studied exhibited selectivity in affecting the EGFR pathway depending on whether EGFR is mutated or wild-type [69]. These data suggest that RESV may have a divergent effect on the EGFR pathway and either activate or inhibit it. However, this leads to the questions: What mechanism is responsible for the increase of IL-8 level observed in HCC827 cells after incubation with RESV? Did RESV in these cells alone inhibit the EGFR pathway or enhance its activation, and consequently cause the increase of IL-8 level? To clarify these, some additional studies assessing the activity of the EGFR pathway in the RESV-treated cells are required. Pastore et al. also observed a decrease in IL-8 levels with the use of the EGFR inhibitor PD168393 [69]. In the present study, after using RESV together with PRI-2191, HCC827 cells showed less IL-8 compared to the cells incubated with RESV alone. It is known that 1,25(OH)_2_D_3_ inhibits the EGFR signaling pathway in cancer cells, and perhaps, PRI-2191 could contribute to a decrease in IL-8 levels by inhibiting this pathway in HCC827 cells [70,71,72].

The discovery of mechanisms behind the cancer-immune escape has revolutionized the research on new approaches of anticancer treatments, while the use of immune checkpoint inhibitors has brought new hopes for successful treatment of various cancers. One such immune checkpoint is the PD-1 (programmed death-1) pathway involving the receptor PD-1 (CD279) and ligands PD-L1 (programmed death ligand-1; also known as B7 homolog 1 (B7-H1) or CD274) and PD-L2 (B7-DC or CD273). The binding of PD-L1 to the inhibitory checkpoint molecule PD-1, found on activated T cells, B cells, and myeloid cells, results in anergy and apoptosis of T cells and thus helps the tumor cells to evade the antitumor immunity [73]. It is known that PD-L1 is highly expressed in cancer cells of different origins, including lung cancer cells [74]. Therefore, we analyzed the impact of PRI-2191 in combination with RESV on PD-L1 expression in lung cancer cells. As presented here, PRI-2191 caused significant upregulation of the PD-L1 expression in HCC827 and NCI-H358 cells, while a significant increase in PD-L1 expression was seen in A549 cells only when PRI-2191 was used together with RESV. Moreover, PRI-2191 in combination with RESV significantly increased PD-L1 expression compared to PRI-2191 alone in NCI-H358 cells. Dimitrov et al. showed that vitamin D acts as a direct inducer of the PD-L1 and PD-L2 expression in epithelial and myeloid cells (PD-L2 expression only in myeloid cells). They also characterized VDREs present in both genes and showed that pretreatment of epithelial cells with vitamin D inhibited the activation of CD4+ and CD8+ T cells. Based on these findings, the authors concluded that vitamin D may represent a double-edged sword in controlling the inflammatory immune response [75]. In Crohn’s disease patients, vitamin D treatment also increased PD-1 expression in CD4+CD25+int T cells and reduced T cell activation. On the other hand, in vitro treatment with vitamin D reduced PD-1 expression, thus indicating that the response of T cells to vitamin D stimulation may differ depending on the environmental conditions [76]. In turn, an increase in PD-L1 expression was observed followed by RESV treatment in breast and colorectal cancer cell lines. The authors suggested that the increase of PD-L1 expression caused by priming with or coexposure to stilbenoids, such as RESV, may sensitize the cancer cells to anti-PD-L1 therapy; however, this approach seems controversial [77]. On the other hand, Verdura et al. recently published very interesting results concerning the immunomodulatory properties of RESV. They showed that RESV disrupted N-glycan branching and promoted PD-L1 dimerization, thereby impeding the correct localization of PD-L1 to the plasma membrane and preventing the surface interaction of PD-L1 with PD-1, and as a consequence, the compound increased the susceptibility of cancer cells to T cell-mediated cell death [78]. In a study on ovarian carcinoma, RESV was shown to stimulate immunogenic cell death and cause an increase in the number of mature dendritic cells and cytotoxic T cells, while the combinatorial treatment with PD-1 antibody and RESV markedly inhibited tumor growth in vivo [79]. In the present study, we analyzed the level of PD-L1 expression in the total cell lysates of RESV- and PRI-2191-treated lung cancer cells. Therefore, the question is whether, despite the enhanced upregulation of PD-L1 in the tested lung cancer cells, PD-L1 was retained in the cytoplasmic compartments and not found on the plasma membrane.

## 4. Materials and Methods

### 4.1. Cell Lines and Culturing Conditions

The following cell lines (mutation status of the main genes is indicated in brackets, according to: https://cancer.sanger.ac.uk/cell_lines, accessed on 28 May 2020) were used in this study: A-427 (wtEGFR, mutKRAS, wtp53), A549 (wtEGFR, mutKRAS, wtp53), Calu-3 (wtEGFR, wtKRAS, mutp53), HCC827 (mutEGFR, wtKRAS, mutp53,), NCI-H1299 (wtEGFR, mutNRAS, nullp53), NCI-H1581 (wtEGFR, wtKRAS, mutp53), NCI-H1703 (wtEGFR, wtKRAS, mutp53), and NCI-H358 (wtEGFR, mutKRAS, nullp53).

A549 cell line was obtained from the European Collection of Authenticated Cell Cultures (Salisbury, UK); A-427, Calu-3, HCC827, NCI-H1581, NCI-H1703, and NCI-H358 cell lines were purchased from the American Type Culture Collection (Manassas, VA, USA); and NCI-H1299 cell line was provided by Professor Zdzisław Krawczyk from Cancer Center and Institute of Oncology (Gliwice, Poland). A-427 cells were cultured in Eagle’s medium (PChO IIET PAS, Wroclaw, Poland), supplemented with 10% fetal bovine serum (FBS), 2 mM L-glutamine, 1% amino acid, and 1 mM sodium pyruvate (Sigma-Aldrich, Steinheim, Germany). A549 cells were cultured in Ham’s F-12K (Kaighn’s) Medium (Life Technologies Limited, Paisley, UK), supplemented with 10% FBS (GE Healthcare, Logan, UT, USA). Calu-3 cells were cultured in Dulbecco’s Modified Eagle’s Medium (Life Technologies Limited, Paisley, UK), supplemented with 10% FBS (GE Healthcare, Logan, UT, USA) and 2 mM L-glutamine (Sigma-Aldrich, Steinheim, Germany). HCC827, NCI-H1299, NCI-H1703, and NCI-H358 cells were cultured in RPMI 1640 + GlutaMAX-I medium (Life Technologies Limited, Paisley, UK), supplemented with 10% FBS (GE Healthcare, Logan, UT, USA). All culture media contained 0.1 mg/mL streptomycin (Sigma-Aldrich, Steinheim, Germany) and 100 U/mL penicillin (Polfa Tarchomin, Warsaw, Poland). Cells were cultured in the AutoFlow Water Jacket Laboratory CO2 incubator (NU–5510 E; NuAire, Plymouth, MN, USA) at +37 °C, in a humidified atmosphere saturated with 5% CO_2_.

### 4.2. Compounds

The vitamin D metabolite (24*R*)-1,24-dihydroxycholecalciferol, (24*R*)-1,24(OH)_2_D_3_, coded as PRI-2191, was synthesized in the Department of Chemistry at the Pharmaceutical Research Institute (PRI) in Warsaw, Poland. Samples of PRI-2191, dried down at PRI from methanol solutions under argon in amber vials, were dissolved in 99.8% ethanol (POCH, Gliwice, Poland) and stored at −20 °C for further analysis. RESV was purchased from Selleck Chemicals (Houston, TX, USA).

### 4.3. Antiproliferative Activity

To determine the antiproliferative activity, the lung cancer cell lines were seeded onto 96-well plates at different densities as follows: 0.5 × 10^3^ cells per well for A549 and NCI-H1299 cell lines and 2.5 × 10^3^ cells per well for the remaining cell lines. After 24 h, PRI-2191 was added to the wells at concentrations of 1000, 100, 10, and 1 nM, and RESV was added at concentrations of 625, 125, 25, and 1 µM for 72 h either alone or in combination with PRI-2191 at a concentration of 100 nM. The antiproliferative activity of the tested compounds was evaluated using the sulforhodamine B (SRB) assay, as described previously [14]. The experiment was repeated at least three times independently. Proliferation inhibition and inhibitory concentration (IC_50_) in each repeat were analyzed using Cheburator 0.4, Dmitry Nevozhay software [80].

### 4.4. Cell Cycle Analysis

For analyzing the progression of the cell cycle, the lung cancer cells were seeded in six-well plates (Corning Inc., Corning, NY, USA) in the culture medium. After incubating the plates for 24 h, the test compounds were added alone and in combination and the cells were exposed to these compounds for 72 h. RESV was used at the concentration of IC_25_–IC_35_ averaged to 20 µM, and PRI-2191 at 100 nM. Then, the cells were collected, washed in phosphate-buffered saline (PBS) (PChO, IIET PAS, Wroclaw, Poland), and fixed in 70% ethanol (POCH, Gliwice, Poland) at −20 °C for at least 24 h. Later, the cells were washed in PBS and incubated at +37 °C for 1 h with RNase (Thermo Fisher Scientific, Waltham, MA, USA). Subsequently, they were incubated with propidium iodide for 30 min (Sigma-Aldrich, Steinheim, Germany) and analyzed using the BD LSR Fortessa flow cytometer with FACS Diva Software (Becton-Dickinson, San Jose, CA, USA) and then using Flowing Software v2.5.1 (University of Turku, Finland). The experiment was done in triplicate.

### 4.5. Caspase-3 Activity

The activity of caspase-3 was analyzed based on its ability to hydrolyze the synthetic substrate Ac-DEVD-ACC, leading to the release of 7-amino-coumarin fluorochrome. The analysis was carried out by measuring the fluorescence intensity with time. Briefly, the lung cancer cell lines were seeded in 24-well plates, and 24 h later, the test compounds were added to the cells, either alone or in different combinations. RESV was used at a concentration of 0–75 µM and PRI-2191 at 100 nM. After incubating the cells for 72 h, caspase-3 activity was measured as described in [19].

### 4.6. Western Blot

For Western blot analysis, the human lung cancer cells treated with PRI-2191 (100 nM) and RESV (100 µM for Calu-3, 20 µM for the remaining cells) for 72 h were collected and lysed in RIPA buffer supplemented with cocktails of protease and phosphatase inhibitors (Sigma-Aldrich, Steinheim, Germany). Protein concentration was determined using the DC Protein Assay (Bio-Rad Laboratories Inc., Hercules, CA, USA). For this purpose, equal amounts of protein in Laemmli sample buffer were first separated in sodium dodecyl sulfate (SDS)-polyacrylamide gels (Bio-Rad Laboratories Inc., Hercules, CA, USA) and transferred to polyvinylidene difluoride membranes (GE Healthcare Europe GmbH, Freiburg, Germany). The membranes were then blocked in 5% nonfat dried milk in Tris-buffered saline (PChO, IIET PAS, Wroclaw, Poland) for 1 h at room temperature. Next, the membranes were incubated overnight at +4 °C with the following primary antibodies in 0.1% PBS-Tween 20 (Tween 20 from Sigma-Aldrich (Steinheim, Germany)): anti-p21, SIRT1 (Cell Signaling Technology, Beverly, MA, USA), anti-CYP24A1, p53, RXRα, VDR, and β-actin (loading control) (Santa Cruz Biotechnology Inc., Dallas, TX, USA). Then, the membranes were incubated with secondary antibodies conjugated with horseradish peroxidase (Santa Cruz Biotechnology Inc., Dallas, TX, USA). Finally, the results were visualized by applying the chemiluminescence method using the ChemiDoc MP Imaging System (Bio-Rad Laboratories Inc., Hercules, CA, USA). The analyses were repeated in triplicates. Densitometric analysis of the Western blots was performed using ImageJ 1.48v software (National Institutes of Health, Bethesda, MA, USA).

### 4.7. qPCR Analysis

For qPCR, the lung cancer cells were first seeded in Petri dishes, and after 24 h, they were exposed to PRI-2191 and RESV, either alone or in combination. After 72 h, the cells were collected from the Petri dishes with TRI Reagent (Sigma-Aldrich, Steinheim, Germany) and kept at −80 °C until further analysis. After thawing, RNA was isolated from all the cell samples using phenol-chloroform extraction [19]. Then, 2 µg RNA of each cell sample was cleaned from genomic DNA using DNase (Thermo Scientific, Vilnius, Lithuania), following which cDNA was synthesized using GoScript kit (Promega, Madison, WI, USA). Four most stable endogenous control genes were chosen from 16 endogenous control candidates by a screening analysis using TaqMan Array Human Endogenous Control Panel array (Life Technologies, Carlsbad, CA, USA). The expression of the chosen genes was analyzed using real-time PCR using TaqMan probes and Master Mix (Life Technologies, Carlsbad, CA, USA) in Viia 7 with Viia 7 Software v 1.1. The following TaqMan probes were used for the study: *18S*rRNA (Hs99999901_s1), *GAPDH* (cat. 4352665), *HPRT1* (Hs99999909_m1), *RPLP0* (Hs99999902_m1), *CD274* (Hs01125301_m1), *CXCL8* (Hs00174103_m1), *RXRA* (Hs01067640_m1), *SIRT1* (Hs01009006_m1), *SPP1* (Hs00959010_m1), *VDR* (Hs01045840_m1), and *VEGFA* (Hs00900055_m1). The ΔΔCT method was used for determining the relative changes in gene expression. The results were analyzed using Expression Suite Software v1.0.3 (Life Technologies, Carlsbad, CA, USA), and the level of gene expression was normalized to the most stable endogenous control.

### 4.8. ELISA Analysis

The secretion of cytokines IL-8, OPN, and VEGF by the lung cancer cell lines treated with PRI-2191 and RESV in conditioned medium was analyzed using ELISA (R&D Systems, Minneapolis, MN, USA). In addition, B7-H1 expression in the tested lung cancer cell lysates was also analyzed using ELISA (R&D Systems, Minneapolis, MN, USA). First, cells were seeded onto culture dishes, and after 24 h, they were exposed to RESV and PRI-2191. After 72 h, the culture media were discarded and the cells were washed with saline, followed by the addition of serum-free and phenol red-free culture medium supplemented with 2 mM L-glutamine (Sigma-Aldrich, Steinheim, Germany) to each well. After incubating the cells for 24 h, the conditioned media were collected and frozen, and the cell number was counted. The concentration of the given growth factors was measured by ELISA, following the manufacturer’s instructions. Then, the level of cytokines in each sample was normalized to the cell number. For ELISA analysis of PD-L1, the lung cancer cell lysates obtained as described in 4.6 were used. The level of PD-L1 in each sample was normalized to the total protein concentration.

### 4.9. Statistical Analysis

Statistical analysis and graph preparation was done using GraphPad Prism 7 (GraphPad Software, Inc., San Diego, CA, USA). Where applicable, one-way ANOVA, and unpaired *t*-test were applied. A *p*-value < 0.05 was considered statistically significant.

## 5. Conclusions

We found that RESV and/or vitamin D derivative may exert a divergent effect on the progression of the cell cycle, the activity of caspase-3, and the expression of several proteins, including p53, VEGF, IL-8, OPN, and PD-L1, depending on the type and genetic profile of cells. RESV augmented VDR expression in a few lung cancer cell lines, which suggests that this compound may influence the vitamin D signaling in lung cancer cells, leading to an increase in vitamin D activity when used in the combination regimens. In addition, when PRI-2191 was combined with RESV, significant upregulation of CYP24A1 expression was noticed in some lung cancer cells, confirming the ability of RESV to modulate the action of vitamin D in these cells. Furthermore, opposite effects were observed on OPN expression in two KRAS-mutant lung cancer cell lines when PRI-2191 was added to RESV. PRI-2191 counteracted the induction of OPN expression in A549 cells but augmented the induction in NCI-H358 cells. On the other hand, only the EGFR-mutant HCC827 cells produced less IL-8 after exposure to RESV together with PRI-2191, compared to the cells incubated with RESV alone. Moreover, PRI-2191 caused significant upregulation of PD-L1 expression in HCC827 and NCI-H358 cells, while a significant increase in PD-L1 expression was seen in A549 cells only when PRI-2191 was used together with RESV. Additionally, PRI-2191 in combination with RESV caused an increase in the level of PD-L1 expression in NCI-H358 cells, compared to PRI-2191 alone. Thus, the study showed that although the impact of vitamin D on cell proliferation or cell cycle was not very significant, it was more pronounced at the molecular level, through the modulation of the expression of several proteins engaged in the regulation of angiogenesis, immune response, and other functions, varied depending on the genetic background of the cancer cells when vitamin D was combined with RESV. We speculate that the differences in response to RESV and PRI-2191 between EGFRmut and KRASmut cell lines may result from the differences in epigenetic modifications since both subtypes are associated with divergent smoking history and smoking can induce epigenetic machinery alterations. In turn, epigenetic changes affect the access of transcription factors, such as VDR, to the genes they regulate. Therefore, the impact of epigenetic changes on divergent activity of RESV and PRI-2191 in lung cancer cells requires additional study.

## Figures and Tables

**Table 1:** Antiproliferative activity of resveratrol (RESV) alone and in combination with (24*R*)-1,24-dihydroxycholecalciferol, (24*R*)-1,24(OH)_2_D_3_ (PRI-2191) on lung cancer cell lines expressed as inhibitory concentration 50 (IC_50_).

	RESV	RESV + PRI-2191 ^1^
**NCI-H1703**	21.6 ± 5.9	17.8 ± 3.3
**NCI-H1581**	31.9 ± 10.1	34.3 ± 11.8
**NCI-H358**	39.8 ± 1.6	47.4 ± 2.9
**A549**	43.9 ± 3.2	36.5 ± 7.4
**NCI-H1299**	52.2 ± 7.0	48.8 ± 8.5
**HCC827**	60.6 ± 12.0	51.9 ± 13.2
**A-427**	60.8 ± 25.0	71.6 ± 35.0
**Calu-3**	231.8 ± 110.3	184.1 ± 64.6

^1^ PRI-2191 in combination with RESV was used at a concentration of 100 nM. Results are expressed as the mean ± standard deviation of at least three independent experiments.

**Table 2:** Proliferation inhibition (%) caused by PRI-2191 on lung cancer cell lines.

	PRI-2191	
	1000 nM	100 nM
**A-427**	3.37 ± 4.91	2.57 ± 3.36
**A549**	2.09 ± 1.38	0.55 ± 1.82
**Calu-3**	0.70 ± 0.99	1.28 ± 0.10
**HCC827**	20.47 ± 6.88	7.52 ± 4.63
**NCI-H1299**	0.61 ± 0.44	0.45 ± 0.54
**NCI-H1581**	3.11 ± 1.50	0.60 ± 0.24
**NCI-H1703**	11.50 ± 5.74	8.82 ± 1.72
**NCI-H358**	* 10.63 ± 0.91	* 12.83 ± 2.69

* The numbers indicate proliferation stimulation.

## Data Availability

Not applicable.

## Supplementary Materials

The following data are available online at https://www.mdpi.com/1422-0067/22/5/2354/s1. Figure S1: Inhibition of cell proliferation in lung cancer cell lines treated with resveratrol (RESV) alone (green) or in combination with (24*R*)-1,24-dihydroxycholecalciferol, (24*R*)-1,24(OH)_2_D_3_ (PRI-2191) (100 nM) (black), Figure S2: Representative histograms of cell cycle analysis of lung cancer cells treated with PRI-2191 (100 nM) and RESV (20 µM), Figure S3: Flow cytometry cell cycle analysis of lung cancer cells after treatment with PRI-2191 and RESV, Figure S4: Induction of caspase-3 activity in lung cancer cells by PRI-2191 and RESV, Figure S5: Western blot analysis of SIRT1 expression in lung cancer cells treated with PRI-2191 (100 nM) and RESV (20 µM), Figure S6: Western blot analysis of (A) p53 and (B) p21 expression in lung cancer cells treated with PRI-2191 (100 nM) and RESV (20 µM), Figure S7: Western blot analysis of (A) CYP24A1, (B) RXRα, and (C) vitamin D receptor (VDR) expression in lung cancer cells treated with PRI-2191 (100 nM) and RESV (20 µM), Figure S8: Analysis of SIRT1, RXRα, and VDR expression in lung cancer cells treated with PRI-2191 (100 nM) and RESV (20 µM) using qPCR, Figure S9: Analysis of vascular endothelial growth factor A (VEGFA) expression in lung cancer cells treated with PRI-2191 (100 nM) and RESV (20 µM) using PCR, Figure S10: Analysis of osteopontin (OPN) expression in lung cancer cells treated with PRI-2191 (100 nM) and RESV (20 µM) using PCR, Figure S11: Analysis of interleukin 8 (CXCL8) (IL-8) expression in lung cancer cells treated with PRI-2191 (100 nM) and RESV (20 µM) using PCR, Figure S12: Analysis of PD-L1 expression in lung cancer cells treated with PRI-2191 (100 nM) and RESV (20 µM) using PCR.
