# Supplementary material for: Differential Response of Lung Cancer Cells, with Various Driver Mutations, to Plant Polyphenol Resveratrol and Vitamin D Active Metabolite PRI-2191
Source: Int J Mol Sci. 2021 Feb 26;22(5):2354. doi: 10.3390/ijms22052354 (PMC7956761; doi:10.3390/ijms22052354)
Supplement: Supplementary file 1 [file ijms-22-02354-s001.pdf]

*Supplementary Material*

# Differential response of lung cancer cells, with various driver mutations, to plant polyphenol resveratrol and vitamin D active metabolite PRI-2191

Ewa Maj, Beata Maj, Klaudia Bobak, Michalina Gos, Michał Chodyński, Andrzej Kutner, and Joanna Wietrzyk

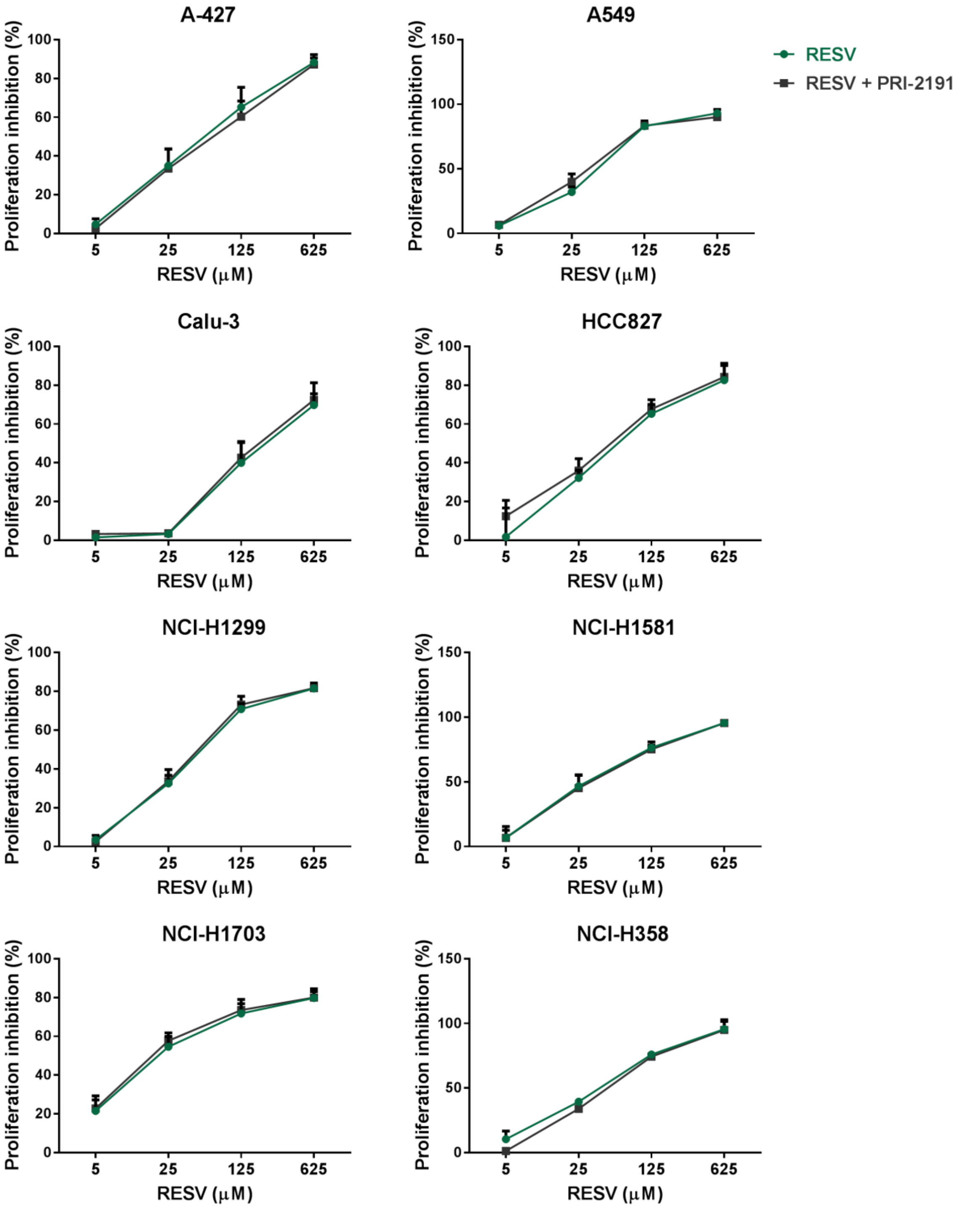


**Figure S1.** Inhibition of cell proliferation in lung cancer cell lines treated with indicated concentrations of RESV alone (green) or in combination with (24*R*)-1,24-dihydroxycholecalciferol, (24*R*)-1,24(OH)2D3 (PRI-2191) (100 nM) (black). Error bars represent the standard error of the mean.

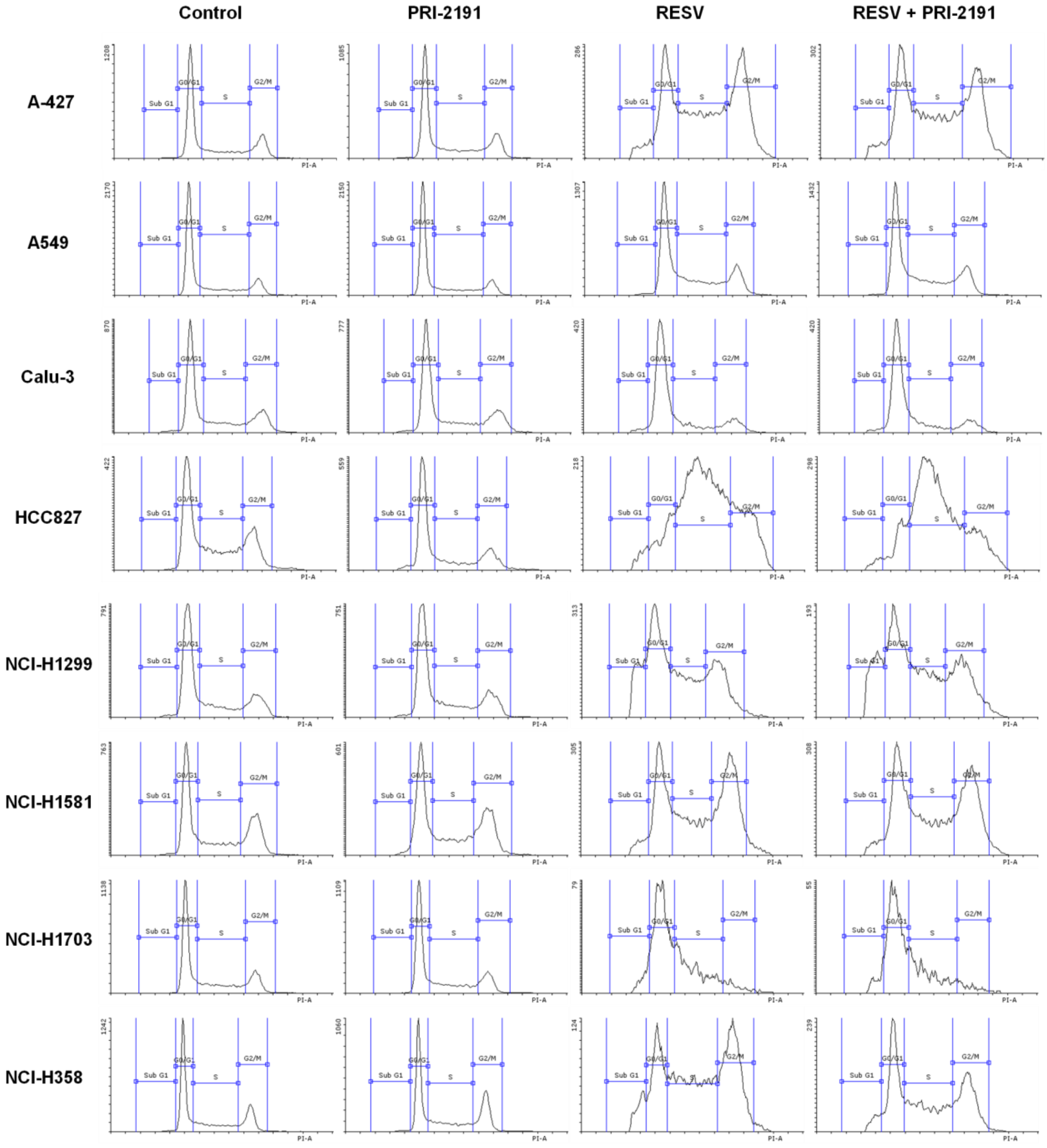


**Figure S2.** Representative histograms of cell cycle analysis of lung cancer cells treated with PRI-2191 (100 nM) and RESV (20 μM). Data were analyzed using Flowing Software v2.5.1.

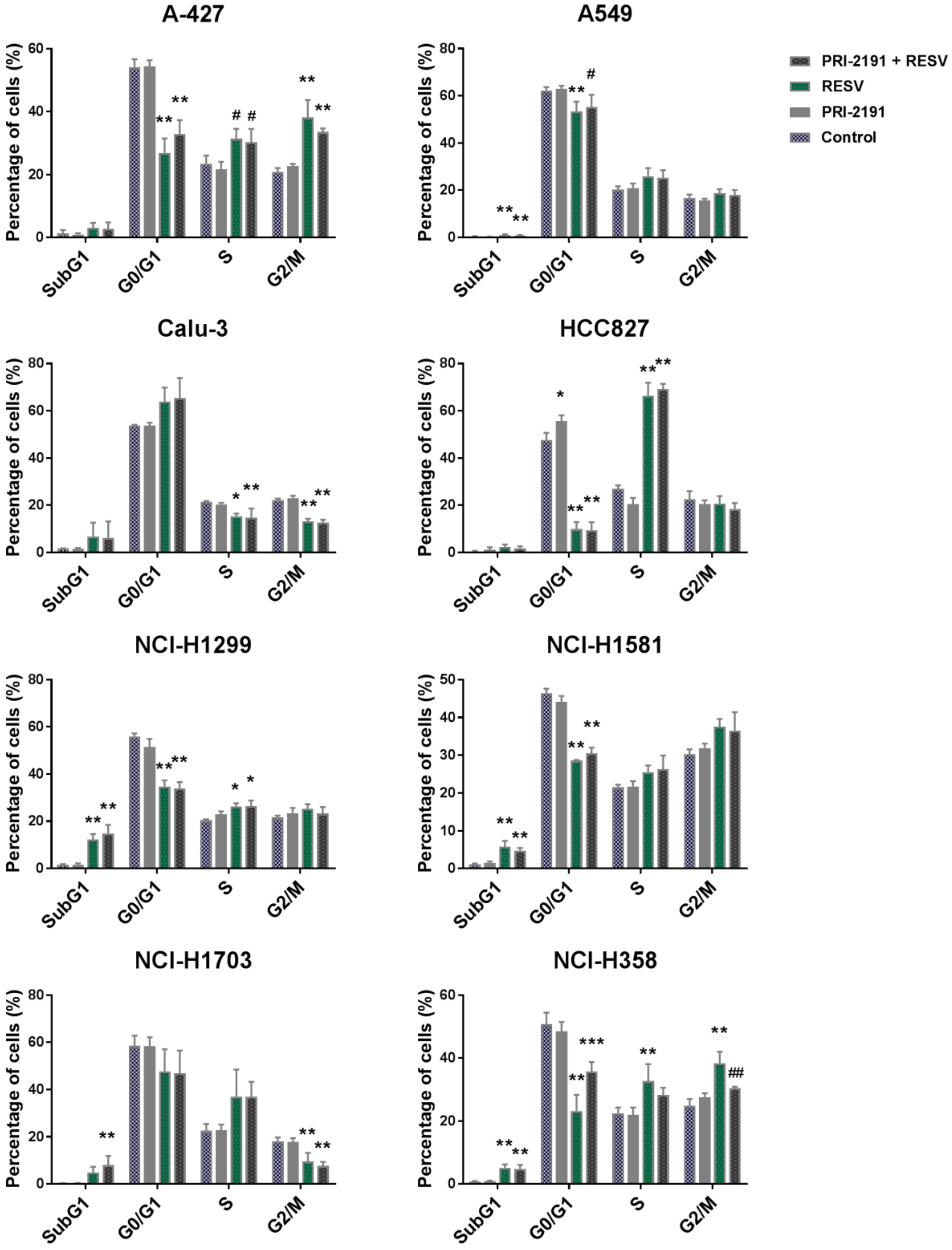


**Figure S3.** Flow cytometry cell cycle analysis of lung cancer cells after treatment with PRI-2191 and RESV. PRI-2191 was used at a concentration of 100 nM and RESV at 20 μM. Data were analyzed using the Flowing Software v2.5.1. *Compared to control (untreated cells); **compared to control and PRI-2191; ***compared to control, PRI-2191, and RESV; #compared to PRI-2191; ##compared to RESV ($p < 0.05$, One-way ANOVA with Tukey's Post-Hoc with multiple comparisons).

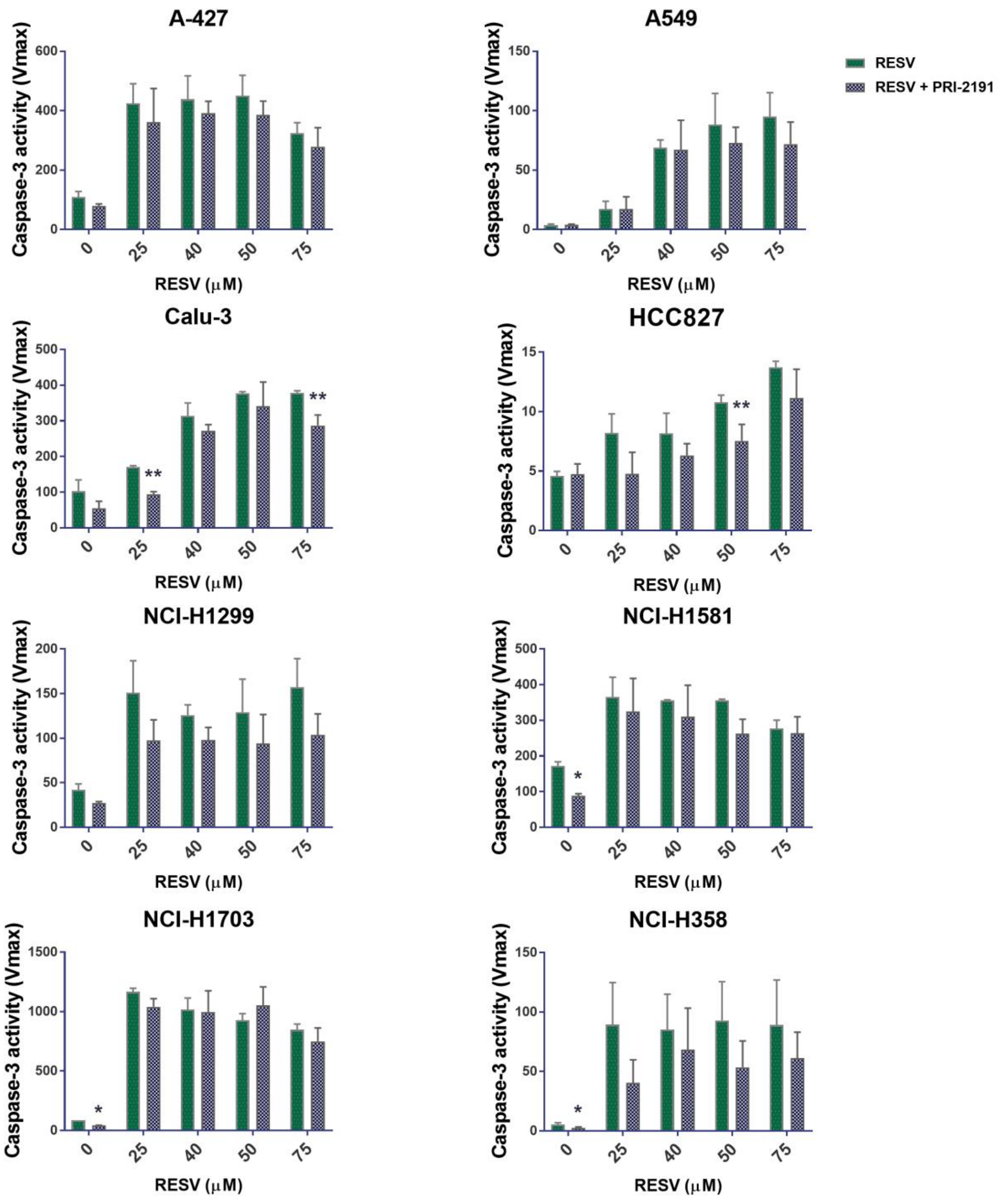


**Figure S4.** Induction of caspase-3 activity in lung cancer cells by PRI-2191 and RESV. RESV was used at a concentration of 0–75 μM and PRI-2191 at 100 nM. Cells were lysed and the substrate (Ac-DEVD-ACC) was added to cell lysates. Fluorescence was measured with time, and kinetics was calculated as RFU/min. Data were analyzed using Gen5 2.09 software. *Compared to control (for PRI-2191 alone); **compared to RESV alone ($p < 0.05$, Student's *t*-test, RESV at the indicated concentration was compared with the corresponding concentration of RESV used with PRI-2191).

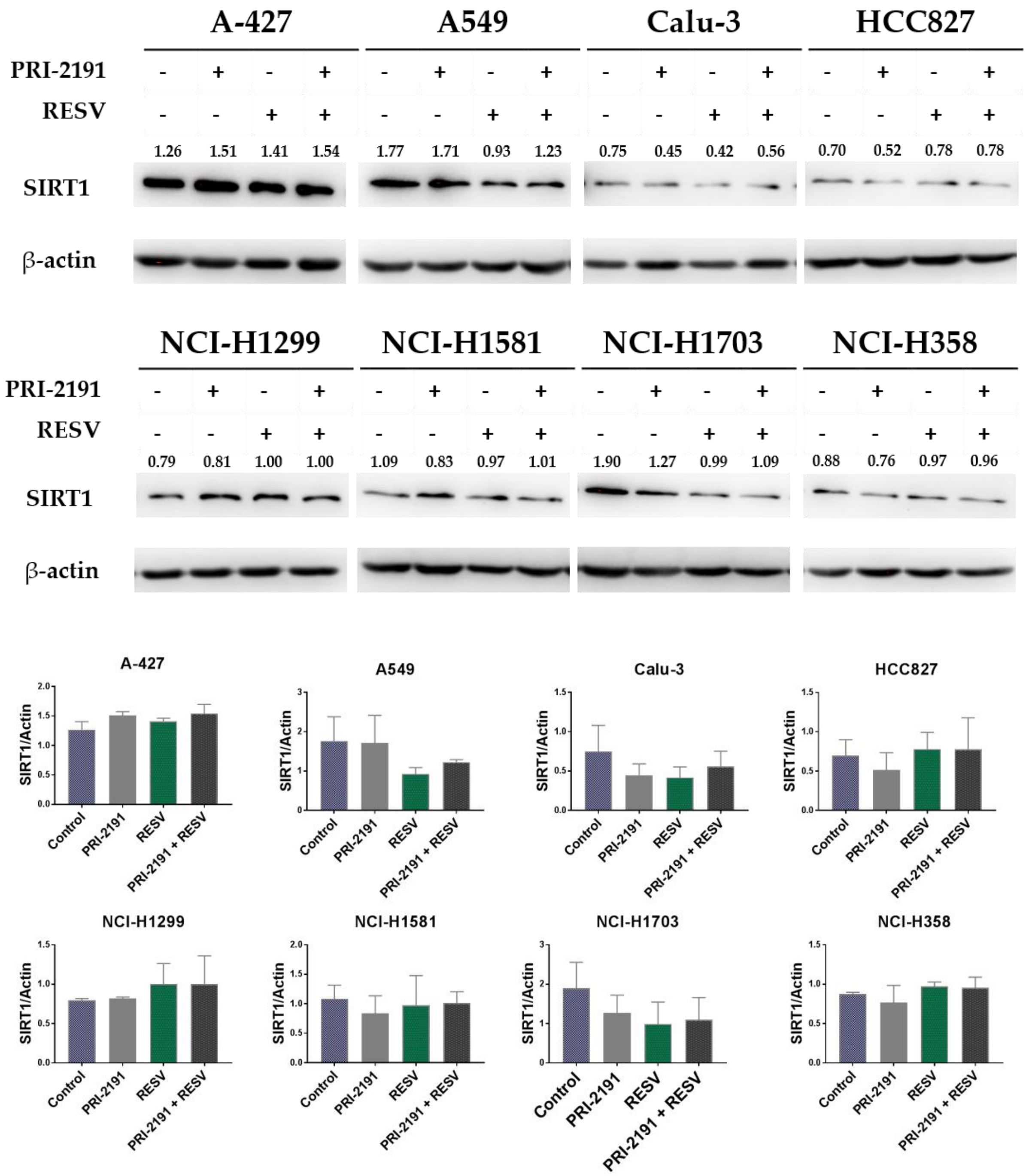


**Figure S5.** Western blot analysis of SIRT1 expression in lung cancer cells treated with PRI-2191 (100 nM) and RESV (20 μM). Cell lysates were subjected to SDS-polyacrylamide gel electrophoresis and analyzed by Western blotting. Actin was used as a normalization control.

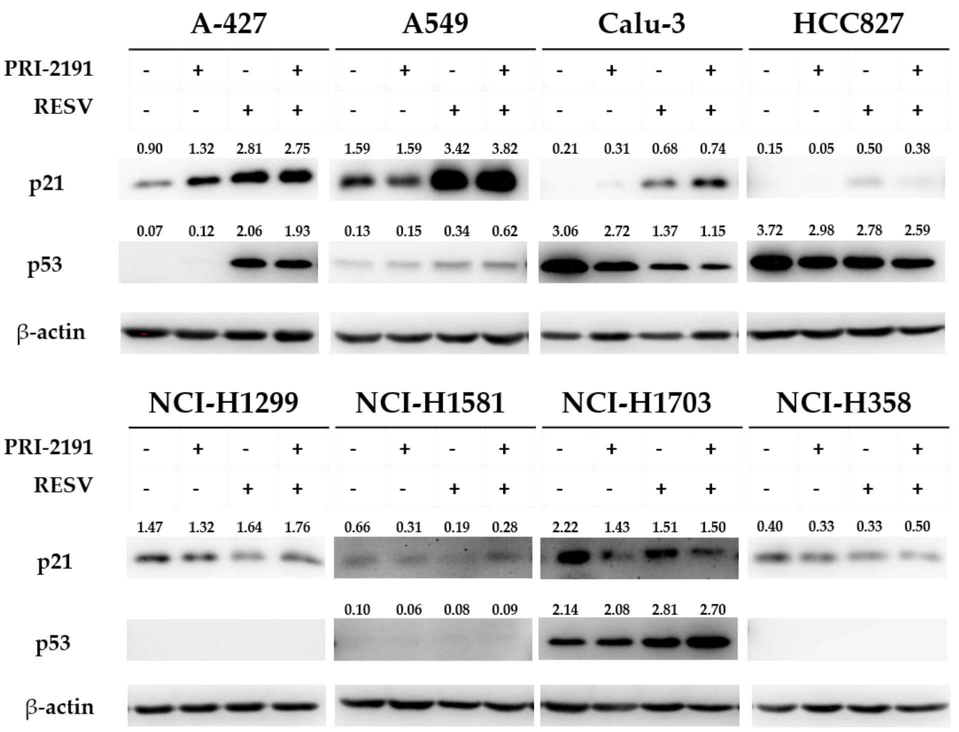
A-427
A549
Calu-3
HCC827
PRI-2191
RESV
0.90 1.32 2.81 2.75
1.59 1.59 3.42 3.82
0.21 0.31 0.68 0.74
0.15 0.05 0.50 0.38
p21
0.07 0.12 2.06 1.93
0.13 0.15 0.34 0.62
3.06 2.72 1.37 1.15
3.72 2.98 2.78 2.59
p53
β-actin
NCI-H1299
NCI-H1581
NCI-H1703
NCI-H358
PRI-2191
RESV
1.47 1.32 1.64 1.76
0.66 0.31 0.19 0.28
2.22 1.43 1.51 1.50
0.40 0.33 0.33 0.50
p21
0.10 0.06 0.08 0.09
2.14 2.08 2.81 2.70
p53
β-actin

**A**

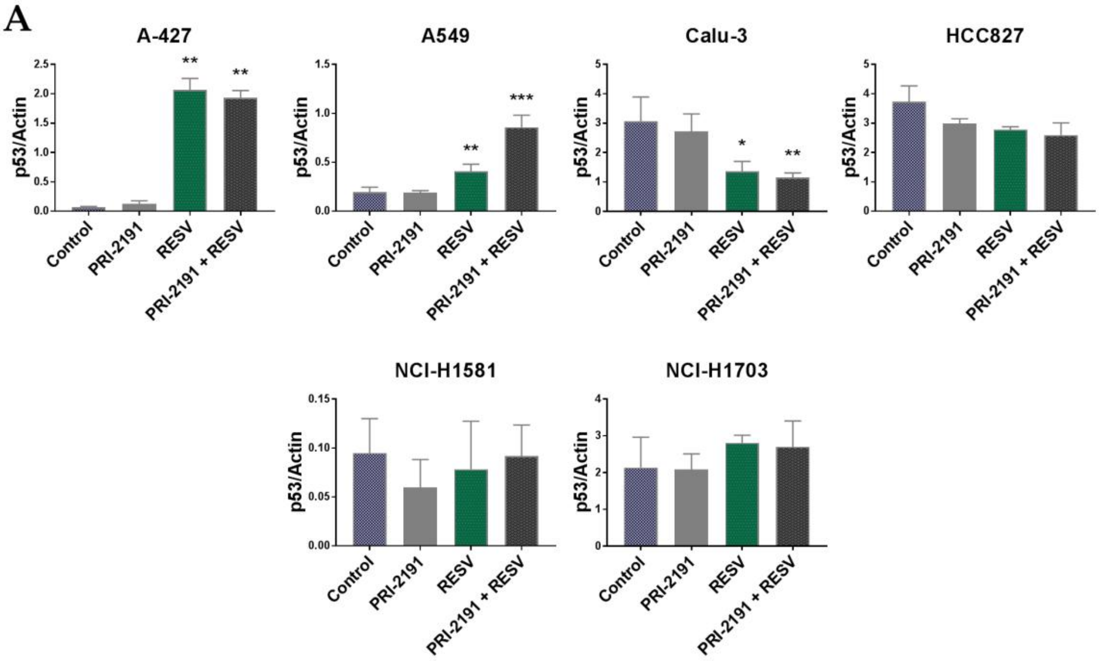
A-427
A549
Calu-3
HCC827
NCI-H1581
NCI-H1703
p53/Actin
Control
PRI-2191
RESV
PRI-2191 + RESV

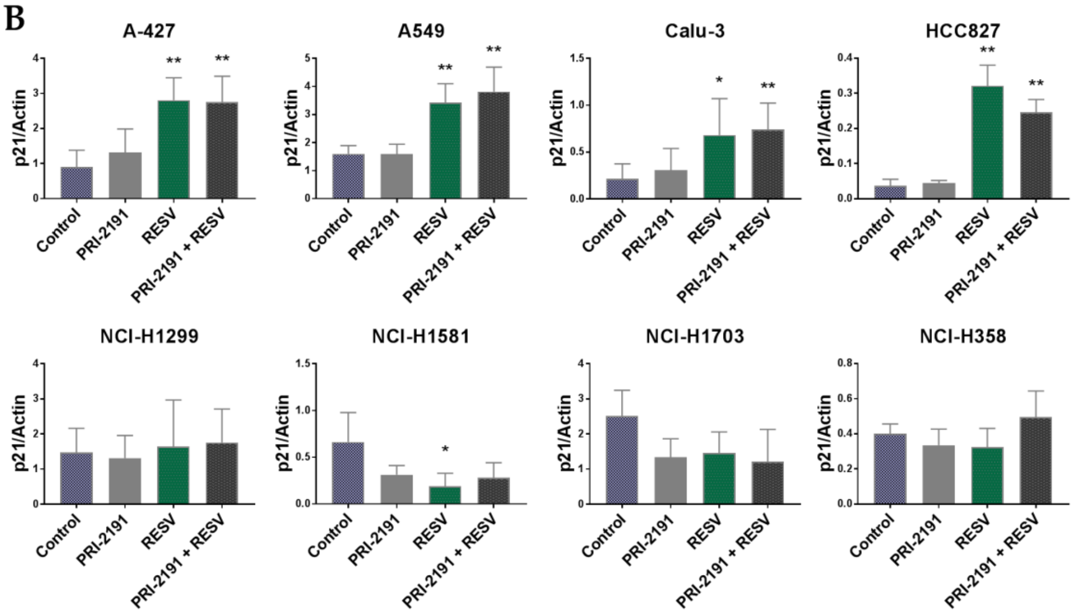


**Figure S6.** Western blot analysis of (A) p53, and (B) p21 expression in lung cancer cells treated with PRI-2191 (100 nM) and RESV (20 μM). NCI-H1299 and NCI-H358 are p53null lung cancer cells. Cell lysates were subjected to SDS-polyacrylamide gel electrophoresis and analyzed by Western blotting. Actin was used as a normalization control. Representative blots are shown. The numbers above blots indicate the mean intensity ratio of the given band normalized to corresponding actin. *Compared to control (untreated cells); **compared to control and PRI-2191; ***compared to control, PRI-2191, and RESV ($p < 0.05$, One-way ANOVA with Tukey's Post-Hoc with multiple comparisons).

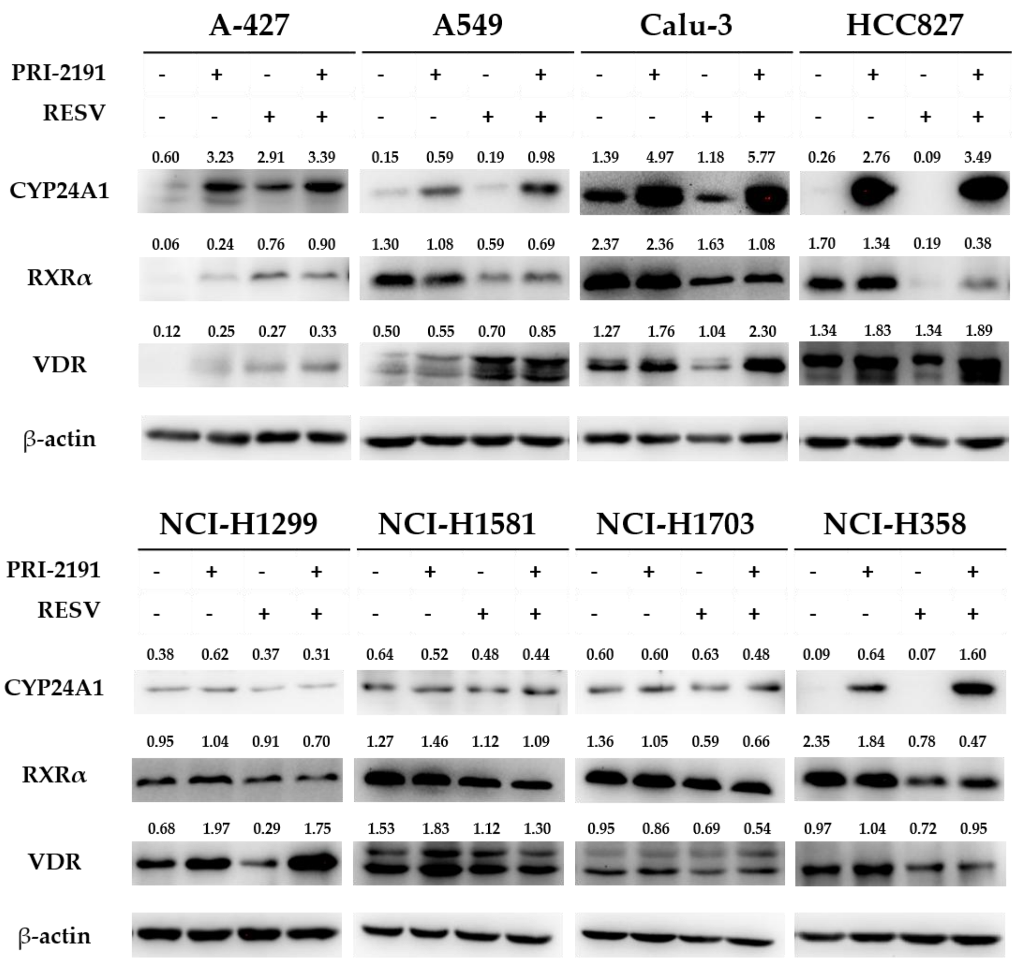
A-427
A549
Calu-3
HCC827
PRI-2191 - + - + - + - + - + - + - + - +
RESV - - + + - - + + - - + + - - + +
0.60 3.23 2.91 3.39 0.15 0.59 0.19 0.98 1.39 4.97 1.18 5.77 0.26 2.76 0.09 3.49
CYP24A1
0.06 0.24 0.76 0.90 1.30 1.08 0.59 0.69 2.37 2.36 1.63 1.08 1.70 1.34 0.19 0.38
RXRα
0.12 0.25 0.27 0.33 0.50 0.55 0.70 0.85 1.27 1.76 1.04 2.30 1.34 1.83 1.34 1.89
VDR
β-actin
NCI-H1299
NCI-H1581
NCI-H1703
NCI-H358
PRI-2191 - + - + - + - + - + - + - + - +
RESV - - + + - - + + - - + + - - + +
0.38 0.62 0.37 0.31 0.64 0.52 0.48 0.44 0.60 0.60 0.63 0.48 0.09 0.64 0.07 1.60
CYP24A1
0.95 1.04 0.91 0.70 1.27 1.46 1.12 1.09 1.36 1.05 0.59 0.66 2.35 1.84 0.78 0.47
RXRα
0.68 1.97 0.29 1.75 1.53 1.83 1.12 1.30 0.95 0.86 0.69 0.54 0.97 1.04 0.72 0.95
VDR
β-actin

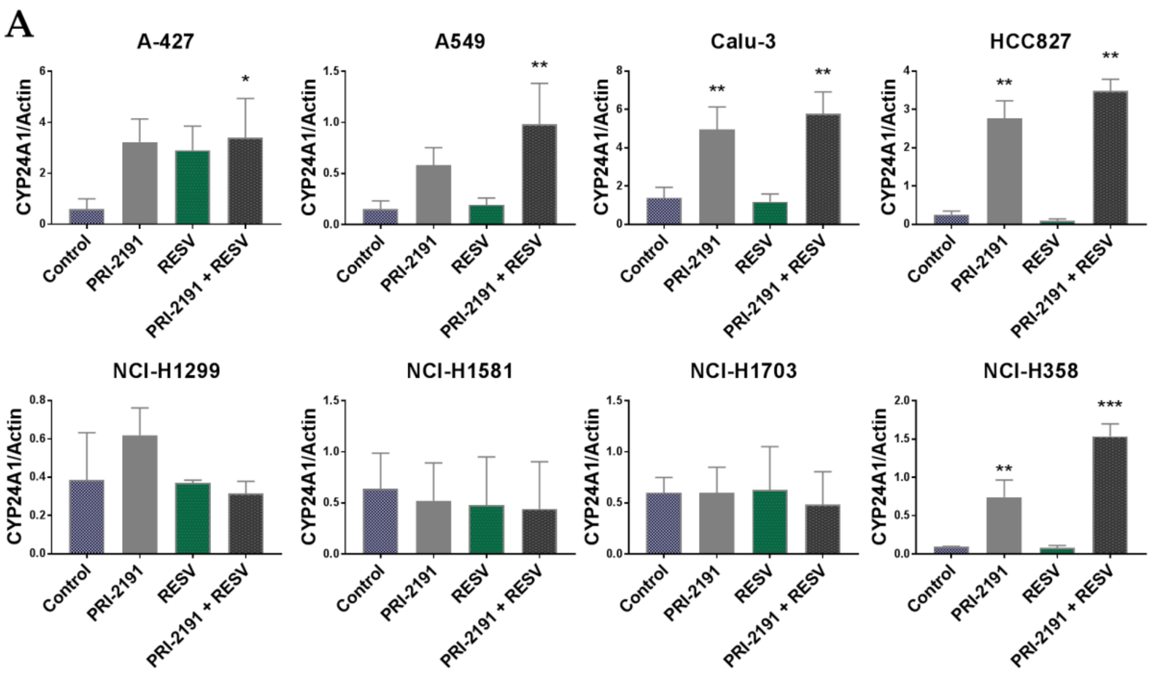
A
A-427
A549
Calu-3
HCC827
NCI-H1299
NCI-H1581
NCI-H1703
NCI-H358
CYP24A1/Actin
Control
PRI-2191
RESV
PRI-2191 + RESV
*
**
***

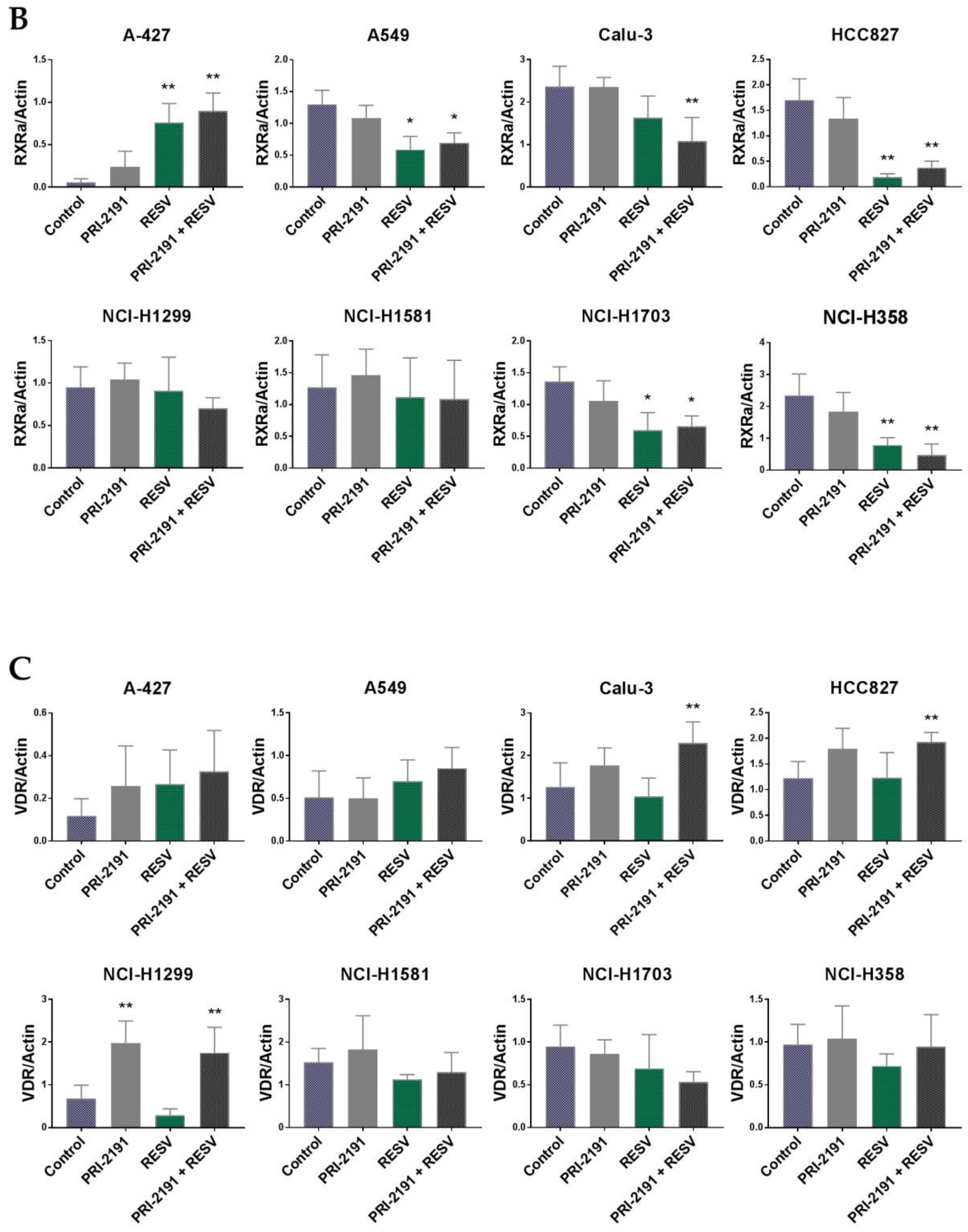


**Figure S7.** Western blot analysis of (A) CYP24A1, (B) RXR$\alpha$, and (C) vitamin D receptor VDR expression in lung cancer cells treated with PRI-2191 (100 nM) and RESV (20 µM). Cell lysates were subjected to SDS-polyacrylamide gel electrophoresis and analyzed by Western blotting. Actin was used as a normalization control. Representative blots are shown. The numbers above blots indicate the mean intensity ratio of the given band normalized to corresponding actin. *Compared to control (untreated cells); **compared to control and RESV (for RXR$\alpha$ compared to control and PRI-2191); ***compared to control, RESV, and PRI-2191 ($p < 0.05$, One-way ANOVA with Tukey's Post-Hoc with multiple comparisons).

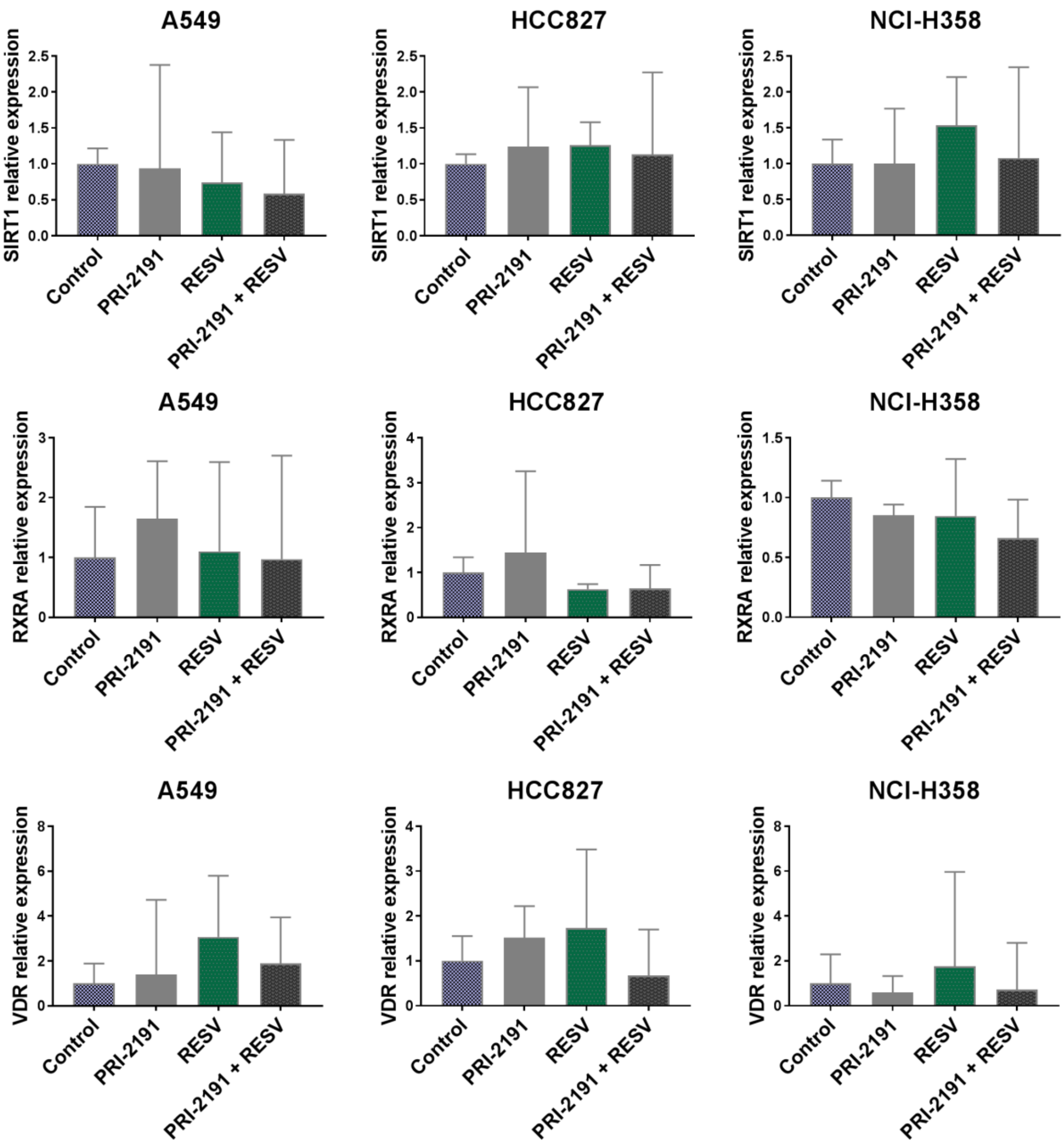


**Figure S8.** Analysis of SIRT1, RXRα, and VDR expression in lung cancer cells treated with PRI-2191 (100 nM) and RESV (20 μM) using PCR. Bars indicate RNA expression normalized to *RPLP0*. Results are expressed as mean RQ ± $RQ_{min}/RQ_{max}$ of at least three independent experiments.

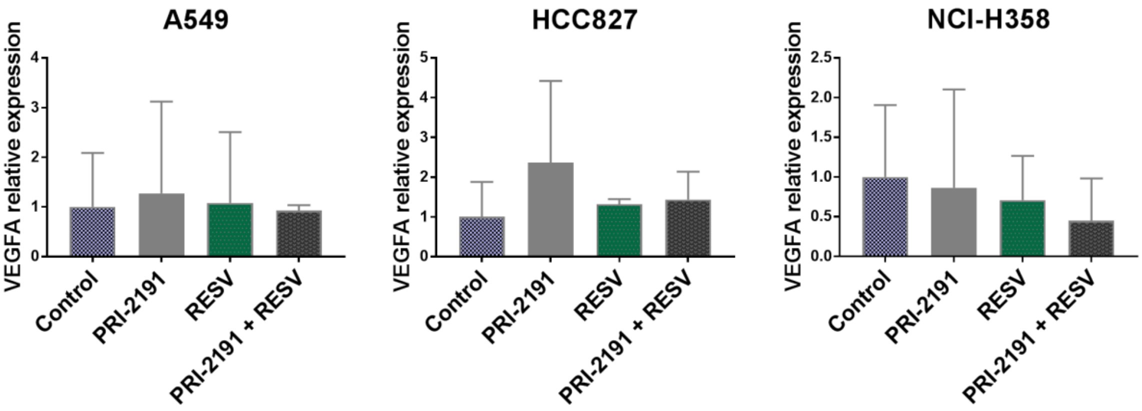


**Figure S9.** Analysis of vascular endothelial growth factor A *VEGFA* expression in lung cancer cells treated with PRI-2191 (100 nM) and RESV (20 μM) using PCR. Bars indicate RNA expression normalized to *RPLP0*. Results are expressed as mean RQ ± $RQ_{min}/RQ_{max}$ of at least three independent experiments. qPCR results: bars indicate RNA expression normalized to RPLP0. Results are expressed as mean RQ ± $RQ_{min}/RQ_{max}$ of at least three independent experiments. A549: *compared to control and PRI-2191; NCI-H358: *compared to control ($p < 0.05$, one-way ANOVA followed by multiple comparisons test).

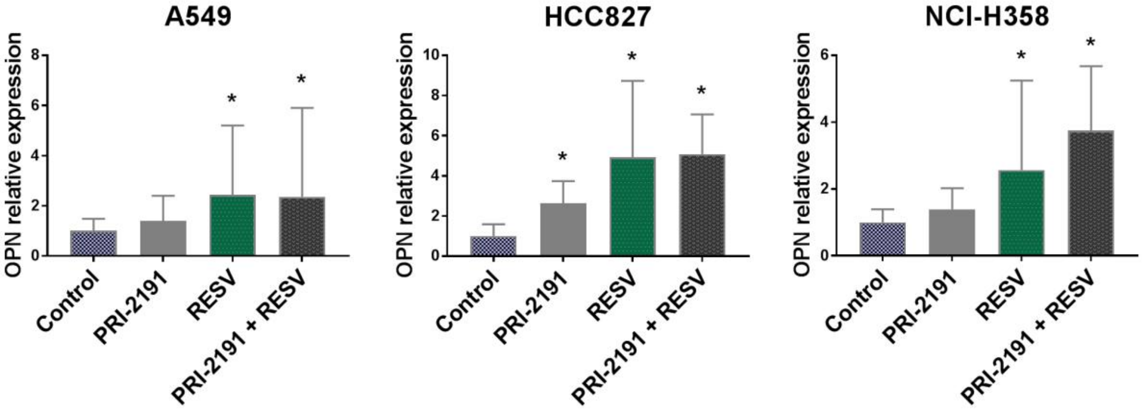


**Figure S10.** Analysis of osteopontin *OPN* expression in lung cancer cells treated with PRI-2191 (100 nM) and RESV (20 μM) using PCR. Bars indicate RNA expression normalized to *RPLP0*. Results are expressed as mean RQ ± $RQ_{min}/RQ_{max}$ of at least three independent experiments. qPCR results: bars indicate RNA expression normalized to RPLP0. Results are expressed as mean RQ ± $RQ_{min}/RQ_{max}$ of at least three independent experiments. *Compared to control; **compared to control and PRI-2191; ***compared to control, PRI-2191, and RESV ($p < 0.05$, One-way ANOVA with Tukey's Post-Hoc with multiple comparisons).

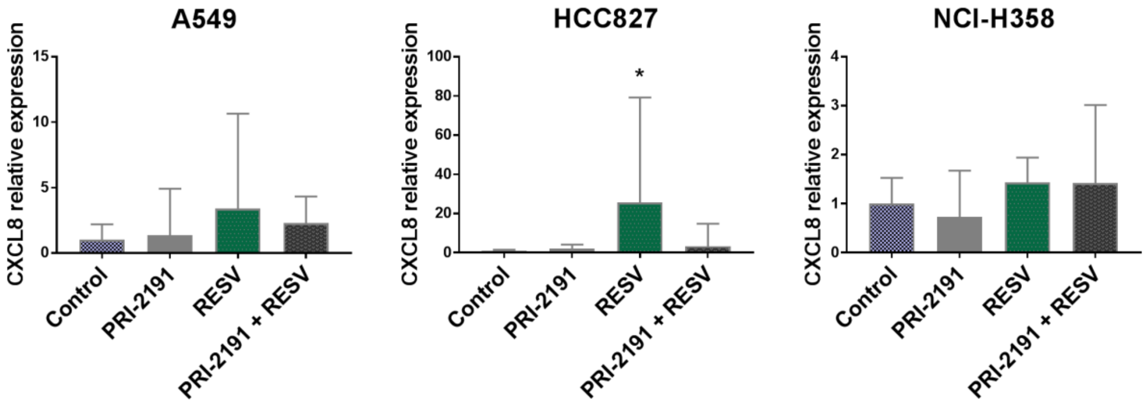


**Figure S11.** Analysis of interleukin 8 (*CXCL8)* (IL-8) expression in lung cancer cells treated with PRI-2191 (100 nM) and RESV (20 µM) using PCR. Bars indicate RNA expression normalized to *RPLP0*. Results are expressed as mean RQ ± $RQ_{min}/RQ_{max}$ of at least three independent experiments. qPCR results: bars indicate RNA expression normalized to RPLP0. Results are expressed as mean RQ ± $RQ_{min}/RQ_{max}$ of at least three independent experiments. *Compared to control; **compared to control and PRI-2191; ***compared to RESV; #compared to PRI-2191 ($p < 0.05$, One-way ANOVA with Tukey's Post-Hoc with multiple comparisons).

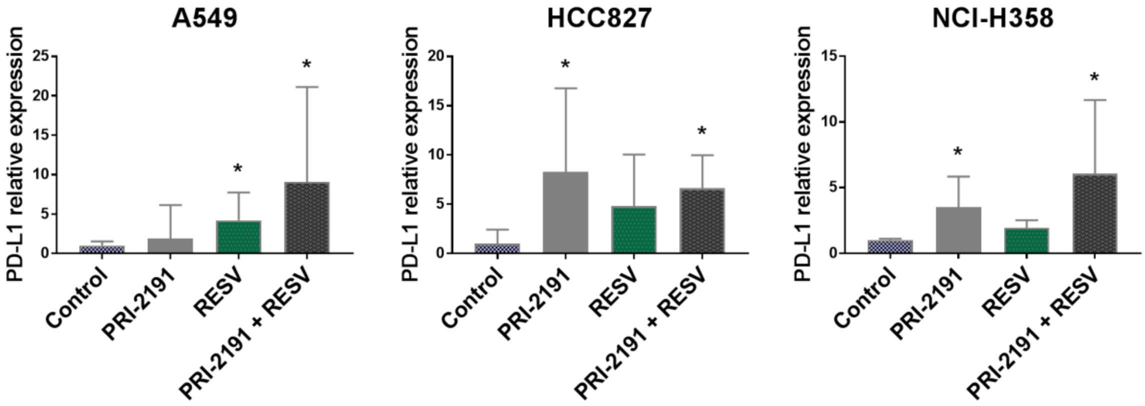


**Figure S12.** Analysis of PD-L1 expression in lung cancer cells treated with PRI-2191 (100 nM) and RESV (20 µM) using PCR. Bars indicate RNA expression normalized to *RPLP0*. Results are expressed as mean RQ ± $RQ_{min}/RQ_{max}$ of at least three independent experiments. qPCR results: bars indicate RNA expression normalized to RPLP0. Results are expressed as mean RQ ± $RQ_{min}/RQ_{max}$ of at least three independent experiments. *Compared to control; **compared to control and RESV; ***compared to control, PRI-2191, and RESV ($p < 0.05$, One-way ANOVA with Tukey's Post-Hoc with multiple comparisons).
